# Gene expression modulation by the linker of nucleoskeleton and cytoskeleton complex contributes to proteostasis

**DOI:** 10.1111/acel.13047

**Published:** 2019-10-01

**Authors:** Amir Levine, Danielle Grushko, Ehud Cohen

**Affiliations:** ^1^ Department of Biochemistry and Molecular Biology The Institute for Medical Research Israel‐Canada The Hebrew University of Jerusalem Jerusalem Israel

**Keywords:** linker of nucleoskeleton and cytoskeleton, neurodegeneration, protein aggregation, proteostasis

## Abstract

Cellular mechanisms that act in concert to maintain protein homeostasis (proteostasis) are vital for organismal functionality and survival. Nevertheless, subsets of aggregation‐prone proteins form toxic aggregates (proteotoxicity) that in some cases, underlie the development of neurodegenerative diseases. Proteotoxic aggregates are often deposited in the vicinity of the nucleus, a process that is cytoskeleton‐dependent. Accordingly, cytoskeletal dysfunction contributes to pathological hallmarks of various neurodegenerative diseases. Here, we asked whether the linker of nucleoskeleton and cytoskeleton (LINC) complex, which bridges these filaments across the nuclear envelope, is needed for the maintenance of proteostasis. Employing model nematodes, we discovered that knocking down LINC components impairs the ability of the worm to cope with proteotoxicity. Knocking down *anc‐1*, which encodes a key component of the LINC complex, modulates the expression of transcription factors and E3 ubiquitin ligases, thereby affecting the rates of protein ubiquitination and impairing proteasome‐mediated protein degradation. Our results establish a link between the LINC complex, protein degradation, and neurodegeneration‐associated proteotoxicity.

## 1 INTRODUCTION

Maintaining protein homeostasis (proteostasis) requires the coordinated activity of a network of quality control and degradation mechanisms (the “proteostasis network”), and is vital for organismal functionality and survival. While the proteostasis network efficiently supervises the integrity of the proteome early in life, its competence declines with age, exposing older organisms to toxic protein aggregation (proteotoxicity) and to the development of a multitude of proteotoxic maladies (Carvalhal Marques, Volovik, & Cohen, 2015). Late‐onset neurodegenerative disorders, such as Alzheimer's disease (AD) and Huntington's disease (HD), are such maladies that are characterized by the accumulation of aberrantly aggregated proteins (Ross & Poirier, 2004).

Several indications highlight the roles of the nucleus in the management of proteostasis. First, cells form quality‐control deposition sites that accumulate aberrantly aggregated proteins within the nucleoplasm (Miller et al., 2015). Secondly, in some cases, aggregated proteins are deposited in juxta‐nuclear quality‐control sites such as aggresomes of mammalian cells. The formation of aggresomes is dependent upon the cytoskeleton, as microtubules sequester aggregated proteins to these sites (Johnston, Ward, & Kopito, 1998). Furthermore, abnormal cytoskeletal components can accumulate in protein deposition sites and underlie pathological aspects of neurodegenerative disorders (Goldman, Yen, Chiu, & Peress, 1983). Thus, the cytoskeleton is essential for protein deposition in quality‐control sites and its stability is associated with proper proteostasis (Hill, Hanzén, & Nyström, 2017).

Nuclear lamins compose the nuclear lamina, a meshwork of intermediate filaments that is a key constituent of the nucleoskeleton, which plays important roles in nuclear architecture and function (Gruenbaum, Margalit, Goldman, Shumaker, & Wilson, 2005). Defects in the nuclear lamina promote aging‐like progeroid phenotypes (Janin, Bauer, Ratti, Millat, & Méjat, 2017), and many studies link the integrity of the nuclear lamina and envelope to proteostasis. For one, the nuclear envelope encompasses a protein quality‐control machinery that mediates the digestion of misfolded proteins (Foresti, Rodriguez‐Vaello, Funaya, & Carvalho, 2014). In addition, the AD‐associated peptide amyloid beta (Aβ) leads to the deformation of the nuclear lamina (Chang et al., 2011) and the expression of a mutated, AD‐linked protein tau causes lamin misregulation (Frost, Bardai, & Feany, 2016). Together, these findings indicate that nuclear components are associated with proteotoxicity, and raise the question of how nuclear components and cytoskeletal elements cooperate to maintain proteostasis.

Microtubules and actin filaments physically interact with nuclear lamins across the nuclear envelope via the linker of nucleoskeleton and cytoskeleton (LINC) complex (Starr & Fridolfsson, 2010). This complex is composed of SUN domain proteins that bind the nuclear lamina and link it to cytoskeletal elements via KASH domain proteins. The LINC complex takes part in transducing mechanical stimuli from the cell exterior to alterations in gene expression (Wang, Tytell, & Ingber, 2009). Although both the cytoskeleton and the nucleoskeleton are linked to aging‐related diseases, it is unclear whether their interactions have a role in the maintenance of proteostasis. To address this, we used the nematode *Caenorhabditis elegans*, which expresses the KASH domain proteins ANC‐1 and ZYG‐12. These proteins interact on one end with actin filaments and the microtubule‐organizing center (MTOC), respectively, and on the other end bind the SUN domain proteins UNC‐84 and SUN‐1, respectively (Starr & Fridolfsson, 2010).

Here, we show that the LINC complex is crucial for proteostasis, as knocking down LINC components enhances proteotoxicity. This effect is dependent upon the gene expression regulatory roles of the LINC complex. Gene expression profiling indicates that knocking down *anc‐1*, which encodes an ortholog of the mammalian proteins nesprin‐1 and nesprin‐2, modulates the expression levels of several T‐box transcription factors and E3 ubiquitin ligases. Functional assays unveiled that the knockdown of *anc‐1* reduces protein degradation by the proteasome.

Together, our results point at the LINC complex as a mediator of proteostasis‐regulating communication between the cytosol and the nucleus, and unveil new roles of transcription factors and ubiquitin ligases in maintaining the integrity of the proteome.

## 2 RESULTS

### 2.1 The LINC complex is required for protection from proteotoxicity but not for lifespan determination

**Figure 1:**
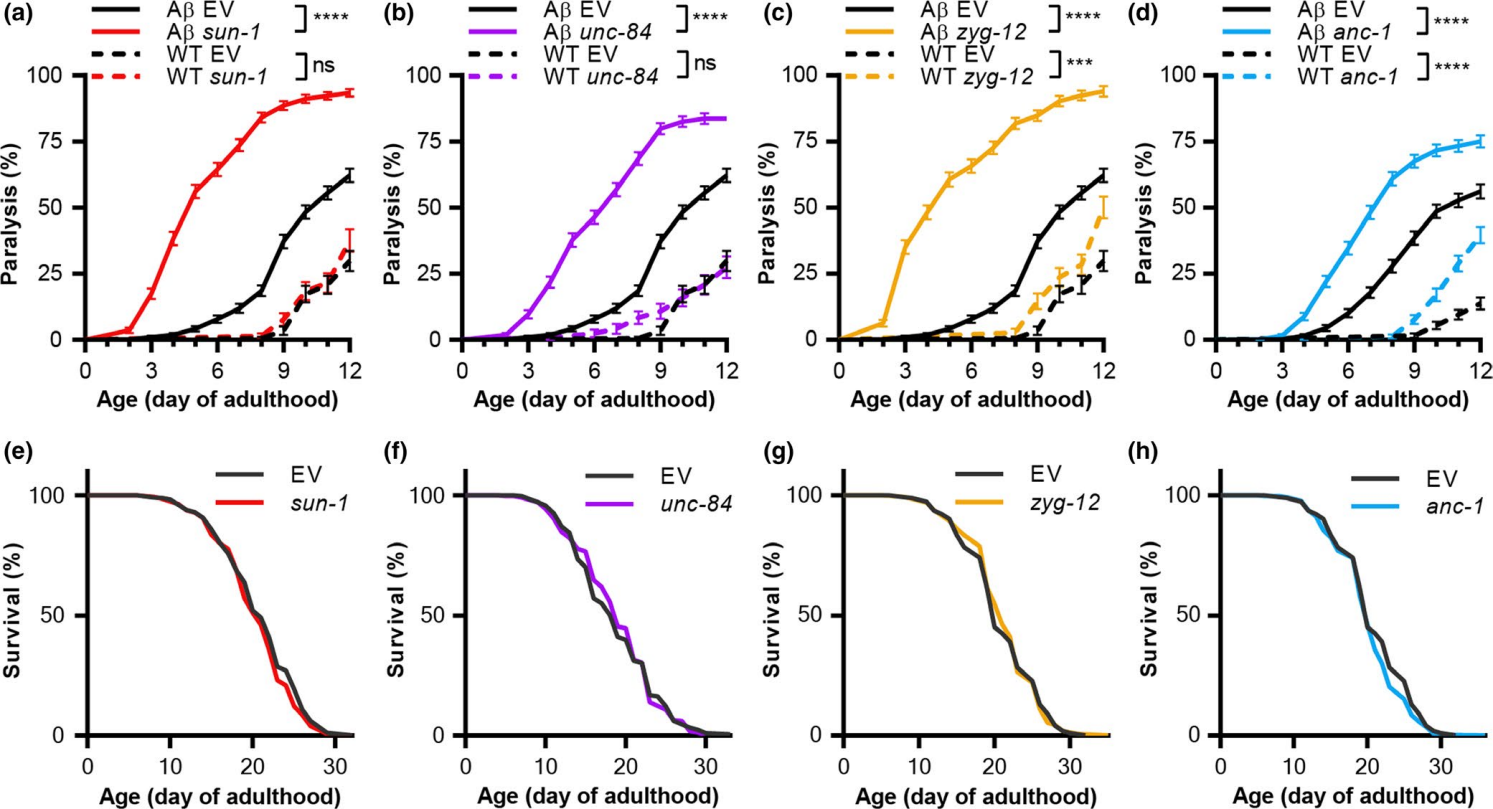
The linker of nucleoskeleton and cytoskeleton (LINC) complex is required for protection from proteotoxicity but not for lifespan determination. (a–d) Paralysis assays of Aβ worms (Aβ, strain CL2006) and of wild‐type animals (WT, strain N2) show that knocking down LINC components enhances Aβ proteotoxicity. In late stages of adulthood, the knockdown of *anc‐1* and of *zyg‐12* enhanced aging‐associated paralysis of WT worms. WT, *n* = 120–240, two independent repeats; Aβ, *n* = 336–362, three independent repeats. (e–h) Survival of temperature‐sensitive sterile animals (strain CF512) shows that LINC component knockdown does not affect lifespan. *n* = 358–361, three independent repeats. Logrank test with a Bonferroni correction. ***p < .001, ****p < .0001. Data are mean ± *SEM*

To test whether the LINC complex is essential to counter proteotoxicity, we employed worms that express the AD‐linked, Aβ_3–42_ peptide in their body‐wall muscles (strain CL2006, hereafter “Aβ worms”). Populations of these animals exhibit progressive paralysis, a phenotype that serves as a measure of Aβ proteotoxicity (Cohen, Bieschke, Perciavalle, Kelly, & Dillin, 2006). Using RNA interference (RNAi), we knocked down the expression of each of the LINC complex genes: *sun‐1*, *unc‐84*, *zyg‐12,* or *anc‐1,* and followed the rates of paralysis within the populations. Our results indicate that the knockdown of any of these genes enhances paralysis, compared to worms that were fed with control bacteria harboring the empty RNAi vector (EV; Figure 1a–d).

To test whether the enhanced paralysis rates observed in the Aβ worms were dependent on Aβ proteotoxicity, we repeated the experiment using wild‐type worms (strain N2). While the knockdown of *sun‐1* and *unc‐84* did not result in paralysis until the end of the experiment, RNAi toward either *anc‐1* or *zyg‐12* enhanced paralysis from day 10 and day 12 of adulthood, respectively. These results imply that ANC‐1 and ZYG‐12 are required for the maintenance of proteostasis in late stages of life.

We also found that knocking down LINC components exclusively during adulthood enhances proteotoxicity (Figure S1a). The lower rate of proteotoxicity, compared with that seen when RNAi treatment was applied from hatching, may stem from a low turnover of the LINC proteins. To examine whether the knockdown of LINC components solely during development affects proteostasis in late stages of life, we treated Aβ worms with LINC RNAi from hatching and transferred them onto *dcr‐1* RNAi on day 1 of adulthood. *dcr‐1* encodes the RNase III enzyme Dicer that is critical for the functionality of the RNAi mechanism. Therefore, the knockdown of *dcr‐1* restores RNAi‐depleted genes to near natural expression levels (Bernstein, Caudy, Hammond, & Hannon, 2001; Dillin et al., 2002). Our results indicate that ANC‐1 and SUN‐1 must be expressed during development to resist Aβ‐mediated proteotoxicity in adulthood; however, this was not the case with ZYG‐12 and UNC‐84 (Figure S1b).

To examine whether the LINC complex is involved in lifespan determination, we treated temperature‐sensitive sterile worms (strain CF512, exhibiting wild‐type lifespan), with RNAi toward either one of the LINC complex components and followed their survival. No change in the lifespans of these populations was observed (Figure 1e–h). These results support the notion that lifespan and proteostasis are separable (Maman et al., 2013).

Aging is accompanied with a decline in the morphology of nuclei; however, this decline is not necessarily coupled with shortened lifespans (Bar & Gruenbaum, 2010; Haithcock et al., 2005). Given the location of LINC on the nuclear envelope, we asked whether the knockdown of the complex components affects nuclear morphology. To address this, we employed transgenic worms that express the nuclear envelope protein emerin (EMR‐1) tagged with GFP. We visualized the nuclei of intestinal cells by fluorescent microscopy and classified them into three categories as defined previously by Haithcock and colleagues (Haithcock et al., 2005): class I, the GFP is smoothly distributed around the nuclear periphery; class II, the nuclear periphery is convoluted, with occasional GFP puncta; and class III, nuclei with intranuclear GFP and decreased peripheral GFP, or with abnormal shapes, stretching, or fragmentation. No observable difference in nuclear morphology can be seen in day 2 and day 6 adult untreated worms compared to animals treated with RNAi toward LINC components. The nuclei of day 10 old animals, on the other hand, deformed slightly more when LINC components were knocked down, most notably when treated with either *sun‐1* or *anc‐1* RNAi (Figure S1c,d).

Since mutations in the human orthologs of *anc‐1*, *SYNE1/SYNE2*, underlie the development of the adult‐onset neurodegenerative disorder spinocerebellar ataxia autosomal recessive 8 (SCAR8) (Beaudin, Gamache, Gros‐Louis, & Dupré, 1993), we focused our efforts on uncovering the mechanism by which ANC‐1 regulates proteostasis. A concurrent RNAi‐mediated knockdown of *anc‐1* and of Aβ reduced the proteotoxic effect of *anc‐1* RNAi compared to worms that were solely treated with *anc‐1* RNAi (Figure S2a). This indicates that the knockdown of *anc‐1* enhances Aβ‐mediated proteotoxicity. To further investigate how ANC‐1 modulates proteostasis, we created an additional RNAi construct that targets the 3′ untranslated region (3′UTR) of *anc‐1.* Although this construct is less efficient (Figure S2b), it is capable of significantly increasing the paralysis rate of Aβ worms without affecting lifespan (Figure S2c,d). Together, these results confirm that ANC‐1 is critically needed to counter Aβ proteotoxicity.

### 2.2 Hyper‐aggregation of polyglutamine and amyloid beta requires ANC‐1

**Figure 2:**
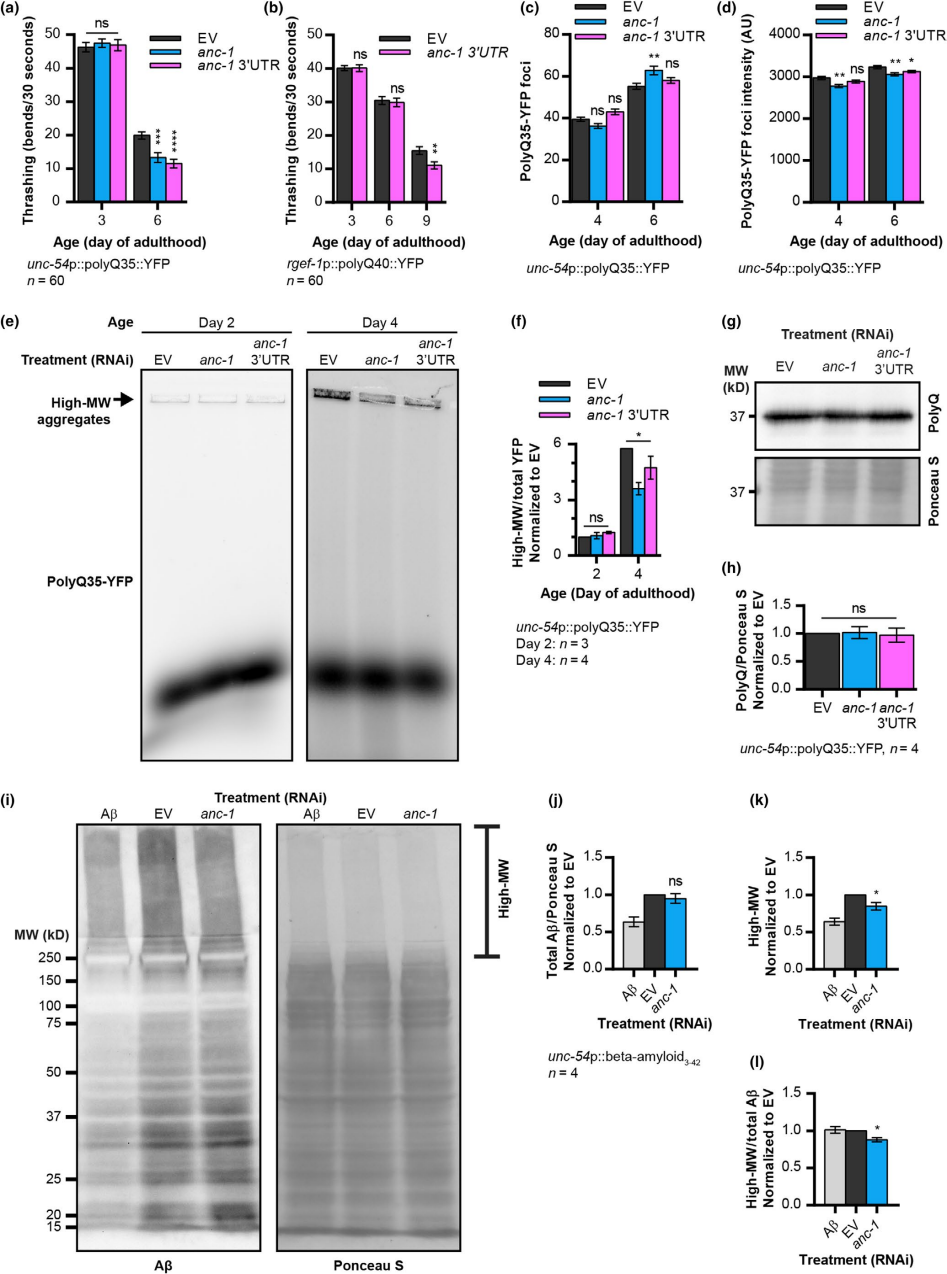
ANC‐1 promotes protective hyper‐aggregation. (a) Thrashing rate of animals that express polyQ35‐YFP in their body‐wall muscles (strain AM140) shows that *anc‐1* knockdown enhances polyQ35‐mediated proteotoxicity in 6‐day‐old worms. *n* = 60, three independent repeats. (b) Thrashing rate of animals that express polyQ40‐YFP in their neurons (strain AM101) shows that in old age (day 9), *anc‐1* knockdown enhances neuronal polyQ40‐mediated proteotoxicity. *n* = 60, three independent repeats. (c) The knockdown of *anc‐1* by RNAi, but not the *anc‐1* 3′UTR RNAi, increases the number of polyQ35‐YFP‐containing foci in body‐wall muscles of 6‐day‐old AM140 worms. (d) The knockdown of *anc‐1* reduces the intensity of fluorescent polyQ35‐YFP‐containing foci in body‐wall muscles of 4‐ and 6‐day‐old AM140 worms. (e,f) PolyQ35‐YFP conformers of AM140 worms were separated by NAGE (e, representative), and high molecular weight (high‐MW) polyQ35‐YFP species were quantified (f). The knockdown of *anc‐1* reduces the formation of high‐MW polyQ35‐YFP aggregates in day 4 old worms. Day 2, three independent repeats; day 4, four independent repeats. (g,h) Western blot of polyQ35‐YFP in day 6 adult AM140 worms (g, representative) and the quantification of four independent repeats (h) show that *anc*‐1 knockdown does not modify total polyQ35‐YFP amounts. Anti‐polyQ antibody, 5TF1‐1C2. (i–l) Western blot of Aβ in day 3 adult CL2006 worms (i, representative) and the quantifications of total Aβ (j) and high‐MW Aβ aggregates (k,l) from four independent repeats show that *anc*‐*1* knockdown reduces Aβ aggregation but does not modify total Aβ levels. Anti‐Aβ antibody, 6E10. One‐way ANOVA (f) with Dunnett's test (a,c,d,h,j–l) and unpaired two‐tailed *t* test (b). *p < .05, **p < .01, ***p < .001, ****p < .0001. Data are mean ± *SEM*

Abnormally long polyglutamine (polyQ) expansions cause multiple neurodegenerative disorders, including Huntington's disease and spinocerebellar ataxias (Ross & Poirier, 2004). To examine whether ANC‐1 is involved in countering proteotoxicity that stems from aggregative proteins other than Aβ, we employed worms that express aggregative polyQ stretches, which are tagged with the yellow fluorescent protein (polyQ‐YFP). Animals that express 35 glutamine repeats tagged with YFP in their body‐wall muscles (polyQ35‐YFP, strain AM140) exhibit an age‐dependent decrease in motility, which can be measured by counting their body thrashes in liquid (Volovik, Marques, & Cohen, 2014). The knockdown of *anc‐1* resulted in decreased thrashing rates by day 6 of adulthood, indicative of proteostasis impairment (Figure 2a). Moreover, *anc‐1* RNAi‐treated worms that express polyQ40‐YFP in their neurons (strain AM101) exhibit reduced thrashing rates by day 9 of adulthood compared with their untreated counterparts (Figure 2b) (Since treating the worms with *anc‐1* 3′UTR RNAi did not induce paralysis of wild‐type worms (Figure S2c), we solely used this construct in the thrashing experiment). Together, our observations indicate that ANC‐1 is critical to resist polyQ proteotoxicity in muscles and neurons.

The sequestration of small, highly toxic oligomers to create large fibrils and their deposition reduces proteotoxicity (Cohen et al., 2006; Shankar et al., 2008). Additionally, the transport of damaged, proteotoxic proteins into inclusion bodies requires functional actin filaments (Hill et al., 2017). These reports raise the prospect that ANC‐1, which interacts with actin filaments, mitigates proteotoxicity by promoting aggregation. PolyQ35‐YFP worms accumulate fluorescent foci in their body‐wall muscles as they age (Morley, Brignull, Weyers, & Morimoto, 2002), which are associated with polyQ35‐YFP aggregation (El‐Ami et al., 2014). Quantifying these foci indicates that the knockdown of *anc‐1* results in no significant change in their numbers in day 4 old worms. Nevertheless, the number of foci was enhanced in day 6 old animals that were treated with *anc‐1* RNAi but not with the *anc‐1* 3’UTR construct (Figure 2c, Figure S3a). Moreover, *anc‐1* knockdown decreases the fluorescent intensity of the foci, suggesting that the polyQ35‐YFP content of these structures is reduced (Figure 2d, Figure S3a). Next, we sought to test whether the knockdown of *anc‐1* affects the rate of polyQ35‐YFP aggregation. To address this, we homogenized polyQ35‐YFP worms, separated the fluorescent conformers using native agarose gel electrophoresis (NAGE), and found that knocking down *anc‐1* reduces the amounts of high molecular weight polyQ35‐YFP aggregates in day 4 old worms (Figure 2e,f). Importantly, we see no difference in total polyQ35‐YFP levels when worms were treated with *anc‐1* RNAi, supporting the notion that ANC‐1 regulates polyQ35‐YFP aggregation, rather than its quantity (Figure 2g,h). Similarly, knocking down *anc‐1* reduces the levels of high molecular weight Aβ species, without affecting total Aβ levels (Figure 2i–l). These results imply that protection against polyQ‐ and Aβ‐mediated proteotoxicity requires ANC‐1, which is conferred, at least partially, by promoting hyper‐aggregation.

Since ANC‐1 is a component of the LINC complex, we asked whether it promotes the accumulation of toxic aggregates in the nuclear vicinity. Using fluorescent microscopy, we observe no accumulation of aggregates around the nuclei of Aβ worms, in both *anc‐1* RNAi‐treated and *anc‐1* RNAi‐untreated animals (Figure S3b,c, Movie S1). Similarly to Aβ worms, polyQ35‐YFP aggregates and nuclei do not co‐localize (Figure S3d, Movie S2). Thus, it is unlikely that ANC‐1 is involved in directing aggregates to a juxta‐nuclear location. To further test whether ANC‐1 promotes hyper‐aggregation as part of the LINC complex, we asked whether the knockdown of the SUN domain protein, UNC‐84, reduces Aβ aggregation. Unlike *anc‐1* RNAi‐treated worms, animals that were treated with *unc‐84* RNAi exhibited no reduction in the quantity of high molecular weight Aβ aggregates (Figure S3e–g, Figure 2i).

### 2.3 ANC‐1 regulates the expression of genes that encode proteostasis‐promoting proteins and transcription factors

**Figure 3:**
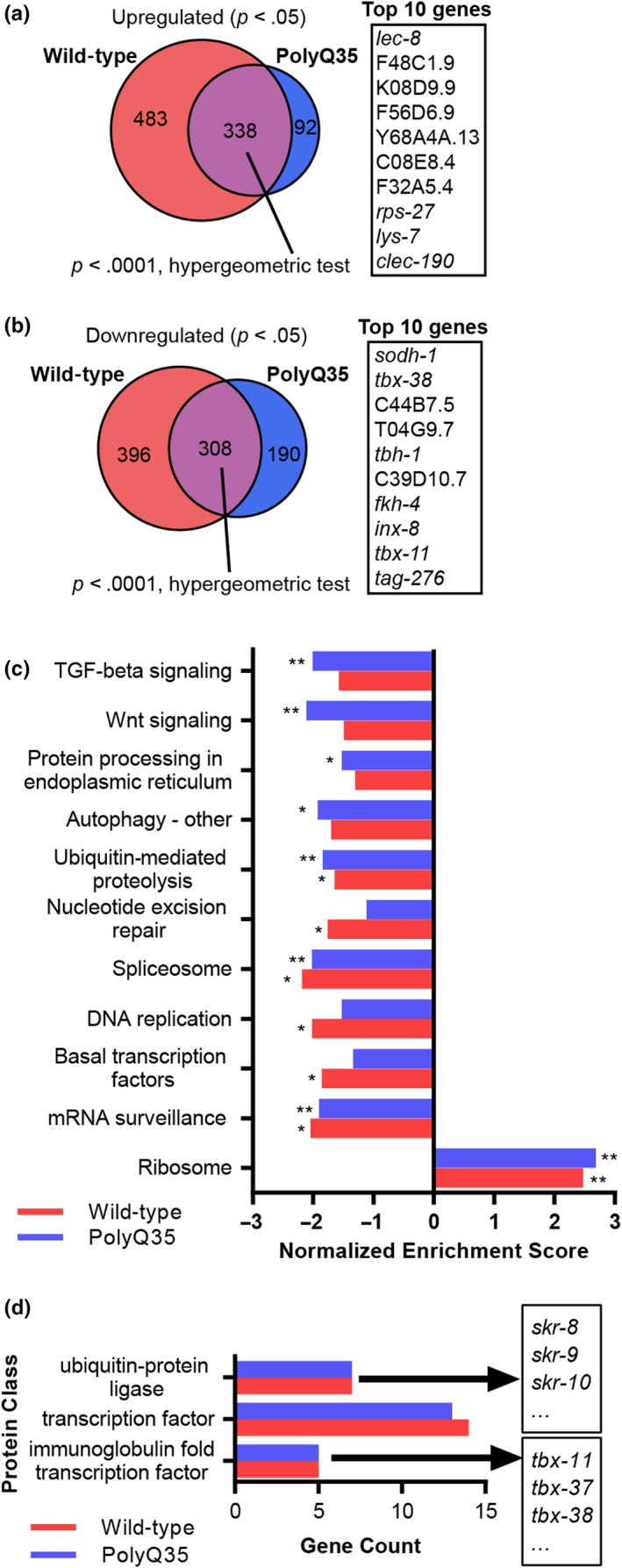
ANC‐1 regulates the expression of genes that encode proteostasis‐promoting proteins and transcription factors. (a,b) Upregulated (a) or downregulated (b) protein‐coding genes of day 6 adult animals show that *anc‐1* knockdown modulates the expression of multiple genes in both wild‐type and polyQ35‐YFP worms. Listed are the 10 genes that exhibited the highest expression fold change in both worm strains. Wild‐type, three independent repeats; polyQ35‐YFP, four independent repeats. (c) Pathway enrichment in *anc‐1* RNAi‐modulated genes based on the KEGG database and gene set enrichment analysis (GSEA). *p < .05, **p < .01. (d) Protein classes from the PANTHER database that are overrepresented in *anc‐1* RNAi‐downregulated genes

Since many components of the proteostasis network are transcriptionally regulated (Carvalhal Marques et al., 2015), and the LINC complex was reported to govern gene expression (Wang et al., 2009), we asked whether ANC‐1 modulates proteostasis by regulating gene expression. We tested this by examining the transcriptomes of wild‐type (N2) and of polyQ35‐YFP animals that were treated from hatching with either *anc‐1* RNAi or left untreated. As we observed increased proteotoxicity by day 6 of adulthood, we harvested the worms at this age (Figure 2a) and measured their protein‐coding transcriptomes by RNA sequencing (RNA‐Seq). The knockdown of *anc‐1* resulted in the upregulation of 338 genes, and the downregulation of 308 genes, in both strains (Figure 3a,b, also see GEO: GSE126585).

One possible explanation to the differential gene expression due to *anc‐1* knockdown suggests that this treatment leads to chromatin reorganization. To test this possibility, we visualized DAPI‐stained nuclei of untreated and *anc‐1* RNAi‐treated day 6 adult worms, and computationally measured their chromatin condensation (Sosnik, Vieira, Webster, Siegfried, & McCusker, 2017). Our results show a potential difference in chromatin organization upon the knockdown of *anc‐1* (Figure S4a,b)*.* This suggests that the robust gene expression modulation we observe after this treatment stems, at least partially, from this chromatin modulation (Figure 3a,b).

An additional possibility is that the knockdown of *anc‐1* modulates gene expression by activating proteostasis‐promoting stress response mechanisms such as the heat shock response (HSR) and the unfolded protein response (UPR) mechanisms of the endoplasmic reticulum (UPR^ER^) and of the mitochondria (UPR^mt^) (Carvalhal Marques et al. 2015). To address whether the knockdown of *anc‐1* activates these mechanisms, we employed worms that express GFP under promoters which are activated by each one of these stress response mechanisms. To detect HSR activation, we used worms that express GFP under the regulation of the *hsp‐16.2* promoter. To follow the rate of UPR^ER^ activation, we utilized animals in which GFP expression is driven by the *hsp‐4* promoter, and nematodes that express GFP under the control of *hsp‐6* promoter were used to measure UPR^mt^. We found no difference in GFP levels between untreated worms and ones that were treated with *anc‐1* RNAi (Figure S5a). Furthermore, the knockdown of *anc‐1* induces no significant change in the expression levels of target genes that are controlled by these stress mechanisms in worms that express either polyQ35‐YFP or Aβ, as measured by our RNA‐Seq and quantitative real‐time PCR (qPCR) experiments (Figure S5b,c). Together, our results show that ANC‐1 is not involved in the regulation of HSR, UPR^ER^, and UPR^mt^.

To characterize biological functions that are regulated by ANC‐1 in our RNA‐Seq data, we used Gene Ontology (GO) annotations and gene set enrichment analysis (GSEA). Our results reveal that multiple biological processes are genetically modified by ANC‐1. One group of genes whose expression is elevated in *anc‐1* RNAi‐treated worms is related to innate immunity (Figure S6). In contrast, genes that are associated with reproduction, vulval development, and embryonic processes exhibit reduced expression levels upon the knockdown of *anc‐1*. In both N2 and polyQ35‐YFP worms, ANC‐1 regulates the expression of genes involved in RNA processing, including transcription, splicing, and translation (Figure S6). Genes related to several processes, including autophagy, are uniquely regulated by ANC‐1 in polyQ35‐YFP worms.

Next, we applied the GSEA algorithm with the KEGG database and discovered that knocking down *anc‐1* lowers the expression of TGF‐β signaling and Wnt signaling‐associated genes in polyQ35‐YFP worms (Figure 3c). In addition, genes associated with autophagy, protein processing in the endoplasmic reticulum (ER), and ubiquitin‐mediated proteolysis exhibit reduced expression levels upon the knockdown of *anc‐1*. Utilizing the PANTHER database's protein class annotations, we found an overrepresentation of ubiquitin‐protein ligases of the *skr* family and transcription factors among the downregulated genes. Specifically, a group of transcription factors named “immunoglobulin fold transcription factor” was most prominent (Figure 3d). In our datasets, this group consists exclusively of T‐box transcription factor‐coding genes (*tbx*).

One possible explanation for the roles of ANC‐1 as a regulator of gene expression suggests that it controls the expression levels of specific transcription factors, which in turn modulate the expression of their target genes. To test this hypothesis, we selected 14 experimentally determined binding motifs of transcription factors that we found to be regulated by ANC‐1*,* from the CIS‐BP database (Weirauch et al., 2014). Then, we examined whether these motifs are enriched in the promoter regions (500 bp upstream and 100 bp downstream of the transcription start site [Niu et al., 2011]), of the *anc‐1* RNAi‐affected genes, and found three such enriched motifs (Table S1). Interestingly, the transcription factors that bind two of these three motifs, TBX‐38 and TBX‐33, are T‐box transcription factors (TBXTFs) that were found to be enriched in the PANTHER analysis.

### 2.4 ANC‐1 enhances the expression of specific T‐box transcription factors to promote proteostasis

**Figure 4:**
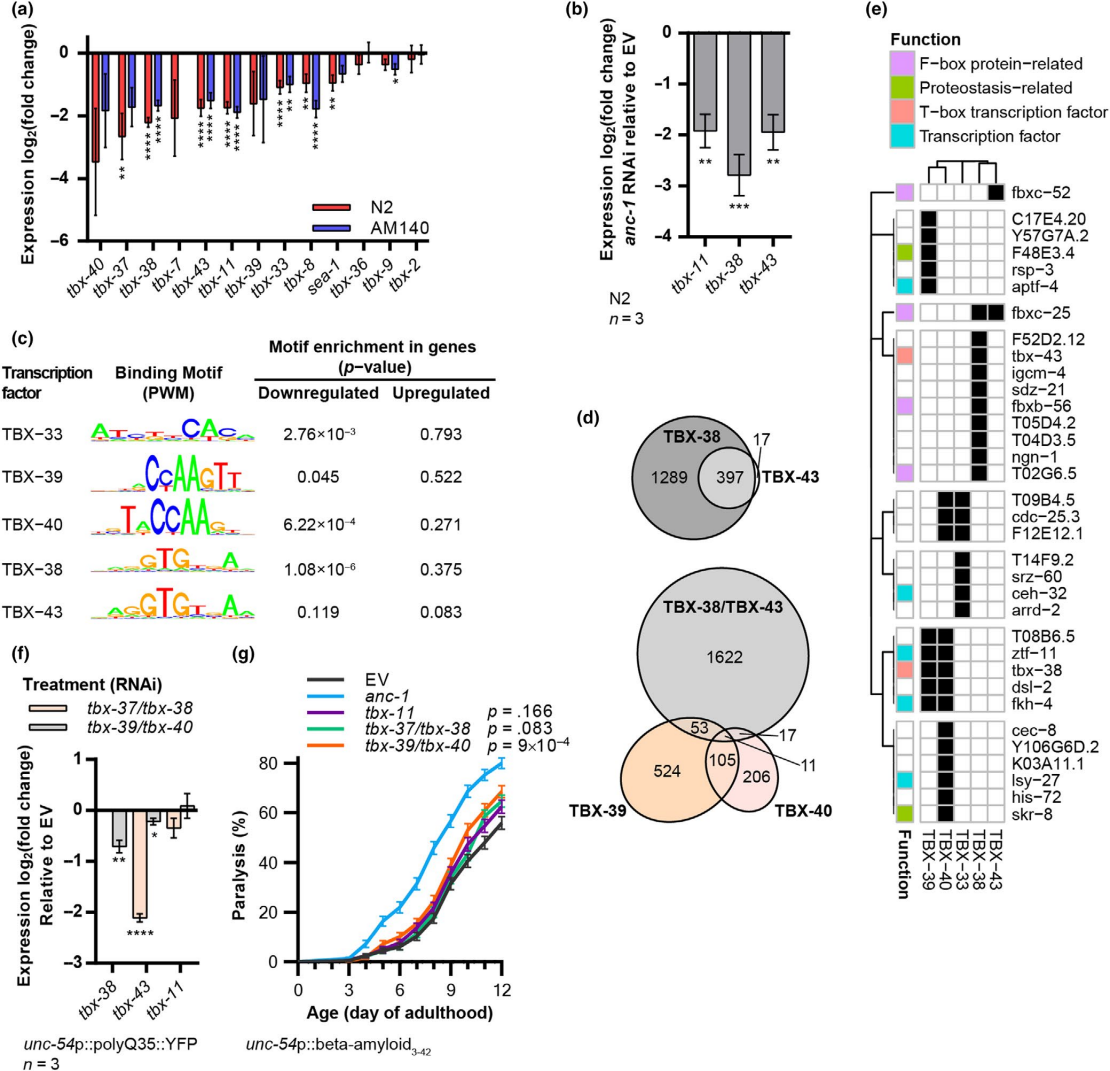
ANC‐1 enhances the expression of specific T‐box transcription factors to promote proteostasis. (a,b) Expression fold change of TBXTF‐coding genes in wild‐type day 6 adult animals, as measured by RNA‐Seq (a) and qPCR (b), shows that *anc‐1* knockdown reduces the expression of multiple TBXTFs. (b) Three independent repeats. (c) Enrichment analysis of TBXTF binding motifs in the promoter regions (500 bp upstream and 100 bp downstream to the transcription initiation site) of ANC‐1‐regulated genes shows that genes downregulated, but not upregulated by *anc‐1* knockdown possess TBXTF binding motifs. (d,e) Predicted TBXTF binding motifs in the promoter regions of all protein‐coding genes of *Caenorhabditis elegans* (d) and genes that are downregulated by *anc‐1* RNAi (e, black cells, *p* < .01) show that target genes of TBX‐38/TBX‐43 and TBX‐39/TBX‐40 are mostly regulated by either TBX‐38/TBX‐43 or TBX‐39/TBX‐40. Function categories refer to the functional association of genes based on GO annotations. (f) Expression fold change of TBXTF‐coding genes in day 6 adult polyQ35‐YFP worms (strain AM140), as measured by qPCR, shows that knocking down TBXTFs reduces the expression of their family members. Three independent repeats. (g) Paralysis of Aβ worms (strain CL2006) shows that knocking down T‐box transcription factors increases Aβ proteotoxicity. *n* = 336–360, three independent repeats. *t*‐test with BH procedure (a), one‐way ANOVA with Dunnett's test (b,f), logrank test with a Bonferroni correction (g). *p < .05, **p < .01, ***p < .001, ****p < .0001. Data are mean ± *SEM*

Since TBXTFs are overrepresented in the list of *anc‐1* RNAi‐regulated genes (Figure 3d) and T‐box binding motifs are enriched in the sequences upstream to ANC‐1‐regulated genes (Table S1), we focused our investigation on this group of genes. *C. elegans* express 22 TBXTFs, which display highly diverse DNA specificity and functions (Okkema, 2017). Our RNA‐Seq experiment indicates that *anc‐1* RNAi lowers the expression of all measured T‐box‐encoding genes (Figure 4a). To validate these results, we selected the three *tbx* genes that showed the most significant effects upon the knockdown of *anc‐1*: *tbx‐11*, *tbx‐38,* and *tbx‐43*, and measured their expression levels using qPCR. Our results confirmed that *anc‐1* RNAi significantly reduces the expression levels of the three tested genes (Figure 4b). Similarly, reduced expression levels were observed in Aβ worms upon *anc‐1* RNAi treatment (Figure S7a). Next, we asked whether the promoter regions of genes that are affected by the knockdown of *anc‐1* contain known TBXTF binding sites. Thus far, five motifs that are bound by *C. elegans* TBX‐33, TBX‐38, TBX‐39, TBX‐43, and TBX‐40 have been characterized (Narasimhan et al., 2015; Weirauch et al., 2014). Importantly, the expression levels of these five transcription factors were detected in our RNA‐Seq experiment as either significantly downregulated or exhibit a trend of downregulation upon *anc‐1* knockdown (Figure 4a). TBX‐38 and TBX‐43 bind the canonical T‐box binding element, while TBX‐33, TBX‐39, and TBX‐40 bind other motif sequences (the last two share a highly similar motif [Narasimhan et al., 2015]). We used computational tools to search for the five T‐box motifs in the promoter regions of the genes whose expression levels were significantly modulated by the knockdown of *anc‐1* in both wild‐type and polyQ35‐YFP worms, and identified an enrichment of four of the motifs in the promoter regions of downregulated genes, but not in those of upregulated genes (Figure 4c).

Since the binding motifs of TBX‐39 and TBX‐40 are very similar, and those of TBX‐38 and TBX‐43 are nearly identical (Figure 4c), we asked how many genes are co‐regulated by the two pairs of TBXTFs. To address this, we searched for the TBXTF binding motifs in the promoter regions upstream of all coding regions of the *C. elegans* genome. Recognition motifs of both TBX‐38 and TBX‐43 were identified in the promoter regions of 397 genes, a majority of the genes that are predicted to be regulated by TBX‐43. Interestingly, only 81 regions were found to contain either a TBX‐39 or TBX‐40 motif, and a TBX‐38 or TBX‐43 motif, (Figure 4d), suggesting that the two pairs of TBXTFs mainly regulate mutually exclusive genes sets.

To further assess a possible regulatory redundancy between different TBXTFs, we identified the genes whose expression levels are modulated by *anc‐1* RNAi, and whose promoter regions harbor TBXTF recognition sites (Figure 4e, *p* < .01). Among these, we see no overlap in the presence of TBX‐39/40 and TBX‐38/43 binding motifs. In addition, we observed an apparent functional specificity of gene sets that are regulated by each pair of TBXTFs. One such gene set is F‐box proteins, which make up part of the proteolysis‐associated E3 ubiquitin ligase SCF (Skp1‐Cul1‐F‐box protein) complex (Zheng et al., 2002). Promoters of genes that code for F‐box proteins were solely found to contain motifs of either TBX‐38, TBX‐43, or both (Figure 4e). The expression of some F‐box protein‐coding genes is indeed regulated by TBXTFs, as observed by qPCR (Figure S7b).

Interestingly, the recognition sites of TBX‐39 and TBX‐40 were identified in the promoter regions of several genes that code for transcription factors, including *ztf‐11* and *fkh‐4*. This comparison also unveils that *tbx‐38* is predicted to be regulated by TBX‐39 and TBX‐40, while *tbx‐43* is predicted to be regulated by TBX‐38. In *C. elegans, tbx‐39* and *tbx‐40*, as well as *tbx‐37* and *tbx‐38,* are similar paralogs (Okkema, 2017). Thus, RNAi constructs toward one member of each pair are predicted to simultaneously knockdown its paralog. Measuring TBXTFs mRNA levels by qPCR shows that, in accordance with our prediction, worms that were fed with bacteria that express the *tbx‐39/tbx‐40* RNAi exhibit decreased *tbx‐38* levels, and knocking down *tbx‐37*/*tbx‐38* decreases the expression level of *tbx‐43* (Figure 4f). Collectively, these results suggest that ANC‐1 controls the expression of TBXTFs that regulate the expression of a range of transcription factors, which include their own family members.

To test whether the TBXTFs are involved in the regulation of proteostasis, we preformed paralysis assays using Aβ worms. Knocking down both *tbx‐39* and *tbx‐40* slightly but significantly enhances Aβ‐mediated proteotoxicity (Figure 4g). The concurrent knockdown of *tbx‐11* or *tbx‐37* and *tbx‐38* resulted in a nonsignificant trend of increased proteotoxicity. One explanation to this mild increase in paralysis compared to the knockdown of *anc‐1* may stem from our observation that *anc‐1* RNAi leads to the simultaneous reduction in a range of TBXTFs (Figure 4a). This decrease in the expression of multiple TBXTFs may culminate to a greater proteostasis impairment.

### 2.5 ANC‐1 modulates ubiquitination and proteasome activity

**Figure 5:**
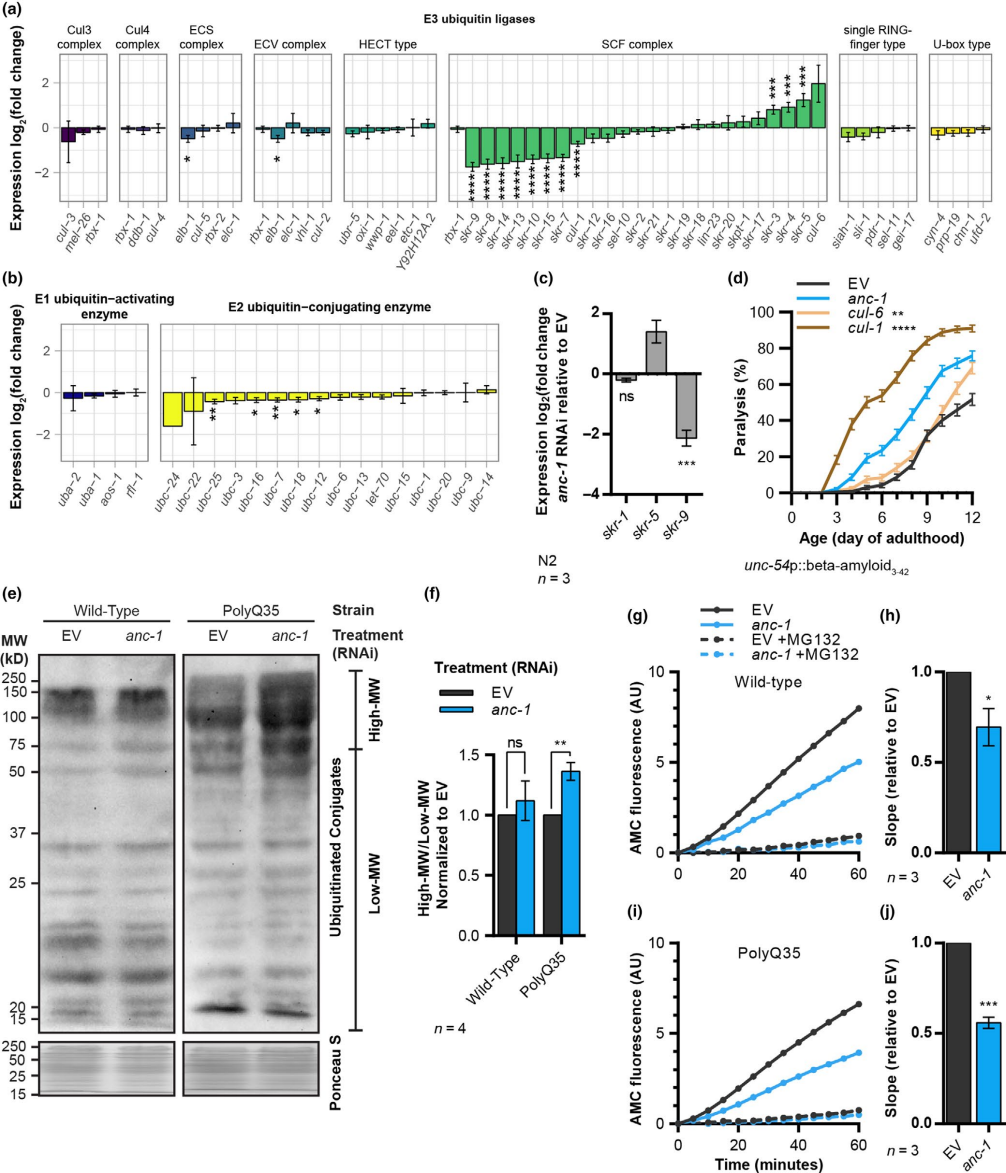
ANC‐1 modulates ubiquitination and proteasome activity. (a–c) Expression fold change of genes that code ubiquitin ligases in day 6 old wild‐type animals, as measured by RNA‐Seq (a,b) and qPCR (c), shows that *anc‐1* knockdown modulates the expression of SCF complex ubiquitin ligase components. Genes that are contained in the “Ubiquitin‐mediated proteolysis in *Caenorhabditis elegans*” entry in the KEGG pathway database were considered as ubiquitin ligases. Three independent repeats. (d) Paralysis of Aβ worms (strain CL2006) shows that the knockdown of *cul‐1* enhances Aβ proteotoxicity. *n* = 217–241, three independent repeats. (e,f) Western blot of mono‐ and poly‐ubiquitinated proteins (e) and the quantification of high‐MW ubiquitin conjugates relative to low molecular weight (low‐MW) conjugates (f) indicate that *anc‐1* knockdown increases the amount of poly‐ubiquitinated proteins in worms that express polyQ35‐YFP (strain AM140), but not in wild‐type worms. Four independent repeats. Anti‐ubiquitin conjugates antibody, FK2. (g–j) Representative in vitro proteasome chymotrypsin‐like activity of day 6 adult worm homogenates as measured using the fluorogenic substrate Suc‐LLVY‐AMC in wild‐type (g) and polyQ35‐YFP (i) worms, and the combined slopes of three independent repeats for each strain (h,j) show that *anc‐1* knockdown decreases proteasome activity. MG132, proteasome inhibitor control. *t*‐test with BH procedure (a), one‐way ANOVA with Dunnett's test (c), logrank test with a Bonferroni correction (d), unpaired two‐tailed *t*‐test (f,h,j), *p < .05, **p < .01, ***p < .001, ****p < .0001. Data are mean ± *SEM*

Our RNA‐Seq results indicate that ANC‐1 regulates the expression of ubiquitin ligases (Figure 3d), and our computational analysis predicts that TBXTFs regulate the expression of genes involved in proteostasis (Figure 4e). Thus, we further analyzed how the knockdown of *anc‐1* affects the expression levels of genes that code for a breadth of ubiquitin ligases. We found that the most prominently affected group of E3 ubiquitin ligases is the *skr* family, orthologs of the mammalian Skp1 gene (Yamanaka et al., 2002). *anc‐1* RNAi elevates the expression of several *skr* genes and reduces the expression of others (Figure 5a, Figure S8a). Importantly, genes that encode other E3 ubiquitin ligase complexes were not significantly affected by *anc‐1* RNAi. *anc‐1* knockdown moderately downregulates the expression of genes that code for E2 ubiquitin‐conjugating enzymes but not of genes that code for E1 ubiquitin‐activating enzymes (Figure 5b, Figure S8a). *skr* genes are similarly upregulated and downregulated when *anc‐1* is knocked down in Aβ worms (Figure S8b).

The *C. elegans* genome harbors 21 *skr* genes, compared with only one Skp1 ortholog in humans (Yamanaka et al., 2002). Six clades of *skr* genes were defined by phylogenetic clustering, which was based on sequence similarities (Nayak et al., 2002). Our results show that *skr* genes of the same clade similarly respond to *anc‐1* knockdown. The expression levels of the clade members, *skr‐3, skr‐4, and skr‐5,* increase, while those of *skr‐7, skr‐8, skr‐9, skr‐10, skr‐13, skr‐14,* and *skr‐15*, which make up two other clades, are reduced (Figure 5a). The expression levels of members of three other *skr* clades showed no significant differences upon the knockdown of *anc‐1*. qPCR validated the RNA‐Seq results, as *skr‐1* exhibited no difference in expression level, *skr‐5* expression was increased, and that of *skr‐9* was decreased (Figure 5c).

The SCF complex is a cullin‐based E3 ubiquitin ligase. Each SCF complex contains one of the 21 SKR proteins in addition to one of two cullins, CUL‐1 or CUL‐6 (Nayak et al., 2002). Importantly, SKR‐7, SKR‐8, and SKR‐10 only bind CUL‐1, and our RNA‐Seq suggests that knocking down *anc‐1* downregulates the expression of *cul‐1* and of *skr‐7*, *skr‐8*, and *skr‐10* (Figure 5a). Conversely, *cul‐6* appears to be upregulated, as well as the genes that code for its functional partners: *skr‐3*, *skr‐4*, and *skr‐5* (Bakowski et al., 2014). If modulating the activity of the SCF complex by ANC‐1 modifies the integrity of the proteome, we expect the knockdown of *cul‐1* to enhance proteotoxicity. To test this, we cultured Aβ worms on bacteria that express either *cul‐1* or *cul‐6* RNAi and performed a paralysis assay. We found that while the loss of *cul‐1* dramatically increases Aβ‐induced paralysis, treating the worms with *cul‐6* RNAi results in a minor increase in this phenotype (Figure 5d). Simultaneous knockdown of *anc‐1* and *cul‐1* leads to a slight increase in the paralysis phenotype compared with worms that were treated only with *cul‐1* RNAi (Figure S8c). Knocking down TBXTFs does not affect the expression of *cul‐1* or *cul‐6*. These results suggest that unlike F‐box proteins, the Cullin component of the SCF complex is not regulated by TBXTFs (Figures S8d and S7b). The surprisingly large effect of *cul‐1* RNAi on the rate of Aβ‐mediated proteotoxicity may stem from additional roles it has as a regulator of proteostasis that are unrelated to *anc‐1.*

The modulation of the SCF complex by *anc‐1* RNAi has led us to ask whether ANC‐1 modifies protein ubiquitination. Blotting total ubiquitinated proteins revealed that knocking down *anc‐1* elevates the amount of high molecular weight (high‐MW) ubiquitin conjugates in polyQ35‐YFP worms but not in wild‐type animals (Figure 5e,f). These results imply that ANC‐1 modifies protein ubiquitination levels in the face of a proteotoxic challenge.

Does the accumulation of high‐MW ubiquitin conjugates emanate from an impairment of proteasome activity? To examine this possibility, we measured the levels of proteasomal activity in homogenates of day 6 adult wild‐type and polyQ35‐YFP worms using fluorogenic proteasome substrates. Our results evidently show that the knockdown of *anc‐1* reduces in vitro chymotrypsin‐like and caspase‐like proteasome activities in both strains (Figure 5g–j, Figure S8e,f) but probably does not affect trypsin‐like activity (Figure S8g,h). The lower rate of proteasome activity in wild‐type worms is puzzling, as unlike in polyQ35‐YFP‐expressing worms, we detected no accumulation of high‐MW ubiquitin conjugates when treated with *anc‐1* RNAi (Figure 5e). This apparent discrepancy likely results from the expression of polyQ35‐YFP, which disrupts global proteostasis (Gidalevitz, Ben‐Zvi, Ho, Brignull, & Morimoto, 2006), thereby exceeding the worms’ protein degradation capacity. In contrast, in wild‐type worms, the quantity of ubiquitinated proteins is lower, as the protein degradation capacity is sufficient to digest these molecules.

## 3 DISCUSSION

**Figure 6:**
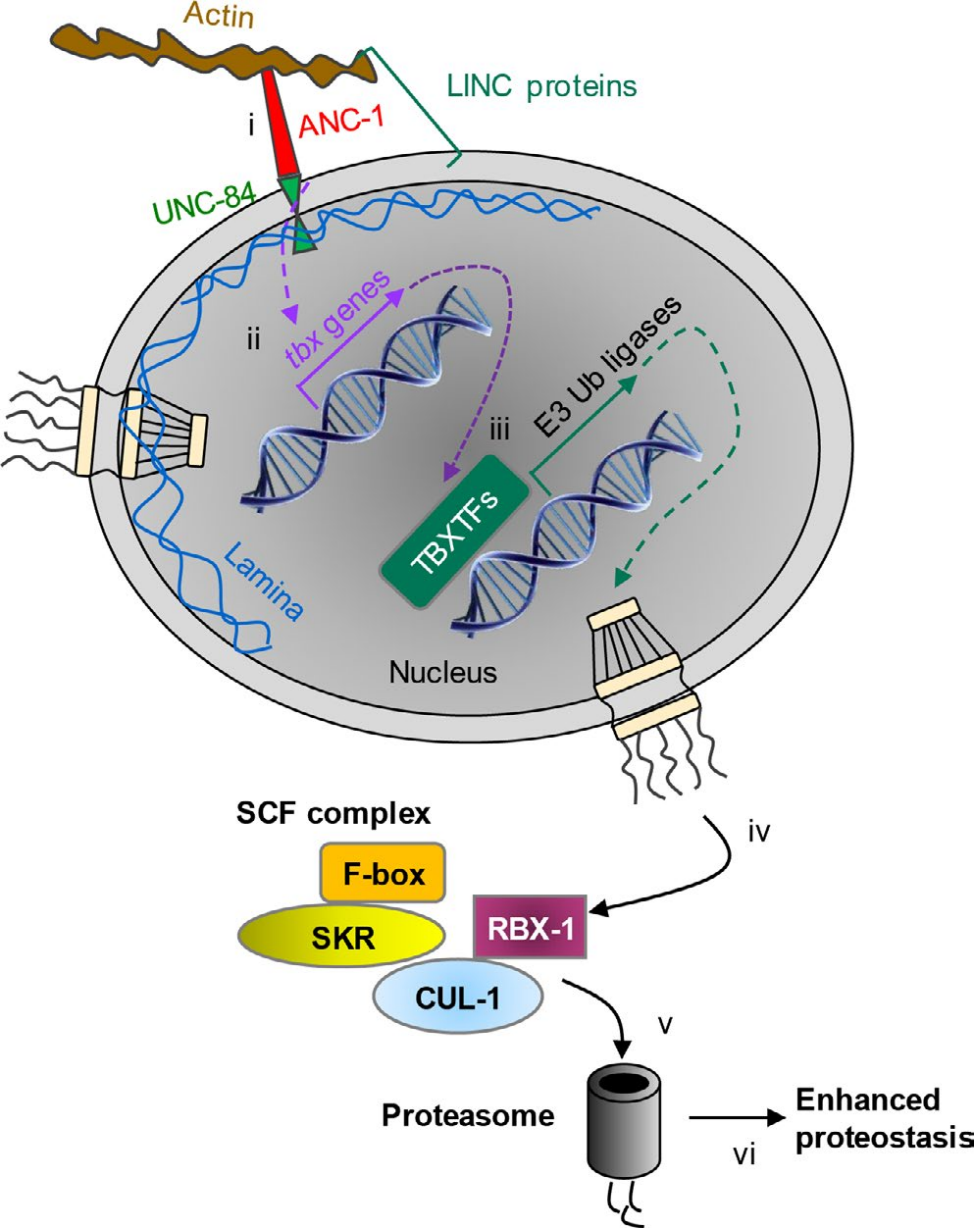
Model of the regulation of proteostasis by the LINC complex. The linker of nucleoskeleton and cytoskeleton complex component ANC‐1 is crucial to cope with proteotoxicity that stems from the aggregative peptide Aβ and polyQ35‐YFP protein. In the presence of proteotoxicity, ANC‐1 upregulates the expression of multiple factors that help to resist proteotoxicity (i). Genes that encode the family of TBXTFs exhibit modulated expression levels upon the knockdown of *anc‐1* (ii). In turn, the TBXTFs regulate the expression of genes, including genes that code for other transcription factors and E3 ubiquitin ligases (iii). Components of the SCF complex are prominent among the T‐box‐regulated genes (iv). Accordingly, the LINC complex plays roles in the regulation of proteasome‐mediated protein degradation (v), thereby contributing to the maintenance of proteostasis (vi)

This study was designed to address the question of whether the cytoskeleton and the nucleoskeleton functionally interact to promote proteostasis. Utilizing model worms, we found that the LINC protein ANC‐1 is vital to cope with proteotoxicity which stems from the aggregative molecules Aβ and polyQ35‐YFP. Using RNA‐Seq and computational tools, we discovered that in the face of proteotoxicity ANC‐1 elevates the expression of multiple factors that confer proteostasis (Figure 6). In particular, genes that encode TBXTFs exhibit modulated expression levels upon the knockdown of *anc‐1*. These TBXTFs are predicted to regulate the expression of genes that code for other transcription factors and components of the SCF complex. Accordingly, our results support the notion that the LINC complex plays roles in the regulation of proteasome‐mediated protein degradation, thereby contributing to the maintenance of proteostasis. Nevertheless, a comprehensive epistatic investigation is needed to fully explore the relations between these LINC‐regulated entities.

### 3.1 LINC‐regulated proteostasis mechanisms

Interestingly, our results (Figure 2) indicate that ANC‐1 confers the hyper‐aggregation of polyQ35‐YFP and Aβ. This protective mechanism (Cohen et al., 2006) is predicted to reduce the levels of highly toxic oligomers by sequestering them to create high‐MW aggregates of lower toxicity (Shankar et al., 2008). One possible explanation of how ANC‐1 promotes hyper‐aggregation suggests that it interacts with cytoskeletal filaments to stabilize protein aggregates and enable their transport to deposition sites. Such sites were shown to accumulate potentially toxic aggregative proteins (Cohen & Taraboulos, 2003; Mishra, Bose, Gu, Li, & Singh, 2003), and were suggested to be protective, at least in early stages of life (Dubnikov, Ben‐Gedalya, & Cohen, 2017). An alternative theme proposes that upon cytoplasmic proteostasis disturbances, the LINC complex transmits signals to the nucleus to promote protective protein hyper‐aggregation by modulating the expression of genes that code for proteins which exhibit aggregase activity (Cohen et al., 2010). This speculation corresponds with the role of LINC proteins in transmitting signals across the nuclear envelope to regulate the chromatin state (Wang et al., 2009).

An important aspect of the link between the LINC complex and proteotoxicity is its roles in the regulation of proteasome activity. Proteasomes are crucial for proteostasis maintenance (Vilchez et al., 2012), and neurodegeneration‐causing aggregates impair proteasome activity (Thibaudeau, Anderson, & Smith, 2018). Our results indicate that knocking down the expression of *anc‐1* impairs at least two features of the ubiquitin‐proteasome system (UPS). First, it controls the expression level of genes that encode components of the SCF ubiquitination complex. Moreover, SCF complex activity was reported to modify polyQ aggregation (Bhutani, Das, Maheshwari, Lakhotia, & Jana, 2012) and to alleviate AD‐associated amyloid formation and synaptic dysfunction (Gong et al., 2010). Secondly, the knockdown of *anc‐1* impairs at least two of the proteolytic activities of the proteasome (Figure 5g–j and Figure S8e,f). These results imply that the integrity of the LINC complex is imperative for proper protein degradation and strongly suggest that it is critical for the prevention of neurodegeneration. Nevertheless, the observations that the levels of polyQ35‐YFP are nearly identical in worms that were treated with *anc‐1* RNAi and their untreated counterparts (Figure 2g,h), and the impairment of proteasome activity in these animals (Figure 5e–j) suggest that another protein degradation mechanism may be involved in the digestion of polyQ35‐YFP.

### 3.2 Gene expression modulation by impairment of the LINC complex

Our computational prediction highlights TBXTFs as regulators of several proteostasis‐maintaining gene groups. These factors modulate the expression of the F‐box protein components of the SCF ubiquitination complex. In addition, *skr‐8*, a Skp1 component, is predicted to be regulated by TBX‐40. It will be interesting to characterize further the specific gene sets that are regulated by these ANC‐1 controlled factors upon proteotoxic challenges.

Transcription factors are another interesting gene group that is predicted to be regulated by TBXTFs. Certain TBXTFs appear to regulate their own family members. For instance, TBX‐39 and TBX‐40 regulate the expression of *tbx‐38*. Similarly, *tbx‐43* is governed by TBX‐38 (Figure 4f). These results suggest the existence of a circuit by which a specific TBXTF can modulate the expression of many genes by regulating the expression levels of its family members. Similarly, TBXTFs are predicted to control the expression levels of additional transcription factors, such as *ztf‐11* and *fkh‐4* (Figure 4e). The relative prominence of ANC‐1 as a regulator of proteostasis may stem from the broader regulated gene network downstream of this LINC protein, compared with those downstream of TBXTFs.

### 3.3 Mutations in nesprins underlie the development of human diseases

Mutations in *SYNE1* and *SYNE2*, that code for the mammalian orthologs of ANC‐1 (nesprin 1 and nesprin 2), are accountable for the development of two diseases: the neurodegenerative disorder SCAR8, which shares similarities with ALS and Emery‐Dreifuss Muscular Dystrophy (Janin et al., 2017). The mechanisms that underlie the development of these pathologies are largely unknown; however, our findings suggest that dysfunctional proteostasis may contribute to their manifestation. Our study raises the interesting prospect that unlike other neurodegenerative disorders, SCAR8 may not emerge due to the aggregation of a specific protein but instead onsets because of aging‐associated proteostasis impairments. According to this hypothesis, nesprin malfunction leads to dysregulation of gene expression, insufficient proteolysis, and subverts proteostasis to initiate the development of the diseases.

## 4 EXPERIMENTAL PROCEDURES

Detailed experimental procedures can be found in the supplemental information.

### 4.1 *Caenorhabditis elegans* and RNA interference

For the experiments conducted in this study, hermaphrodite *C. elegans* were synchronized using hypochlorite and potassium hydroxide, and cultured at 20°C on NG plates with 100 µg/ml ampicillin that were seeded with bacterial cultures. To induce RNAi, the seeded plates were pretreated with 100 mM IPTG.

### 4.2 Paralysis and lifespan assays

On the first day of adulthood, randomly picked animals were transferred onto 60 mm NG‐ampicillin plates seeded with bacteria, 12 animals per plate. Worms were gently tapped with a platinum wire daily. In lifespan experiments, worms that failed to move were scored as dead. Aβ worms that could not crawl away were scored as paralyzed.

### 4.3 Thrashing assay

On each time point, 20 randomly picked animals were sequentially placed in an M9 buffer drop on top of a microscope slide and allowed 30 s of recovery. Afterward, the number of body bends of each worm was counted for the duration of 30 s.

### 4.4 Fluorescent microscopy

For immunofluorescence and nuclear morphology measurement, worms were prepared as described previously (Cohen et al., 2006). Briefly, the worms were prefixed with 4% paraformaldehyde in MRWB (80 mM KCl, 20 mM NaCl, 10 mM EGTA, 5 mM spermidine, 25% methanol). For immunofluorescence, fixated worms were permeabilized with Triton X‐100, incubated with a blocking buffer, and incubated with the primary and secondary antibodies. Fresh n‐propyl gallate supplemented with DAPI was added to the worms right before imaging, as mounting media. For polyQ35‐YFP foci measurement, on the indicated days worms were collected and immobilized in 20 mM sodium azide, and imaged immediately.

### 4.5 Native agarose gel electrophoresis

On each time point, the worms were homogenized at 4°C. For each sample, 100 µg of total protein was loaded onto a 1% agarose gel and ran at 4°C, 40 V for 15 hr. YFP fluorescence intensities in the gels were visualized using a Typhoon FLA 9500 (GE Healthcare).

### 4.6 SDS‐PAGE and Western blot analysis

The worms were homogenized, and their proteins were separated using polyacrylamide gels and transferred onto a PVDF or nitrocellulose membrane. Post‐transfer, total proteins were stained using Ponceau S and imaged. The membranes were then blocked in 5% nonfat milk and exposed to the primary and secondary antibodies. Chemiluminescence was detected and quantified.

### 4.7 RNA isolation and cDNA preparation

The worms were frozen in liquid nitrogen, thawed on ice, and homogenized at 4°C. Total RNA was isolated using NucleoSpin^®^ RNA Kit according to the manufacturer instructions (MACHEREY‐NAGEL, 740955). cDNA was synthesized by reverse transcription using the iScript cDNA Synthesis Kit (Bio‐Rad, 170–8891).

### 4.8 Quantitative real‐time PCR

Quantitative real‐time PCR was performed with iTaq™ Universal SYBR^®^ Green Supermix (Bio‐Rad, 172–5124). Relative expression levels were determined using the −ΔΔCq method.

### 4.9 RNA sequencing (RNA‐Seq)

Isolated RNA samples were prepared and sequenced using the Illumina HiSeq 2500 by the Technion Genome Center (Technion, Haifa, Israel) according to the CEL‐Seq2 protocol. The RNA sequencing data files that were generated in this study are available in the NCBI Gene Expression Omnibus (GEO) under the accession number GEO: GSE126585. Detailed description of computational analyses appears in the supplemental section.

### 4.10 Proteasome activity assay

Worms were homogenized, and 10 µg of total protein per sample was loaded into a 96‐well plate. 25 µM of a fluorogenic substrate was added: Suc‐LLVY‐AMC (chymotrypsin‐like activity), Z‐LLE‐AMC (caspase‐like activity), or Z‐ARR‐AMC (trypsin‐like activity). The reactions were excited at 380 nm, and emission was measured at 480 nm every 5 min for 1 hr at 37ºC. Results at time 0 of each experiment were defined as baseline.

### 4.11 Statistical analyses

The statistical tests used, statistical significance, error bars, and sample sizes can be found in the corresponding figure legends. “Statistically significant” was defined as *p*‐value <.05.

## CONFLICT OF INTERESTS

The authors declare no competing interests.

## AUTHOR CONTRIBUTIONS

AL and EC conceptualized the study, planned the methodology and experimental design, and wrote the original manuscript draft and its revisions. AL performed most of the experimental work, analyzed the experimental data, visualized the results, and curated the RNA‐Seq data in the GEO database. DG performed thrashing and paralysis assays. EC conducted Western blots and supervised the study.

## Data Availability

The RNA sequencing data files that were generated in this study are available in the NCBI Gene Expression Omnibus (GEO) under the accession number GEO: GSE126585. Detailed description of computational analyses appears in the supplemental section.
