# Supplementary material for: Gene expression modulation by the linker of nucleoskeleton and cytoskeleton complex contributes to proteostasis
Source: Aging Cell. 2019 Oct 1;18(6):e13047. doi: 10.1111/acel.13047 (PMC6826161; doi:10.1111/acel.13047)
Supplement: Supplementary file 1 [file ACEL-18-e13047-s001.pdf]

Gene Expression Modulation by the Linker of Nucleoskeleton and Cytoskeleton Complex Contributes to Proteostasis

Supporting Information

Amir Levine, Danielle Grushko, and Ehud Cohen*

Department of Biochemistry and Molecular Biology, The Institute for Medical Research Israel-Canada, The Hebrew University of Jerusalem, Jerusalem 9112001, Israel.

***Corresponding author: ehudc@ekmd.huji.ac.il**

Figure S1

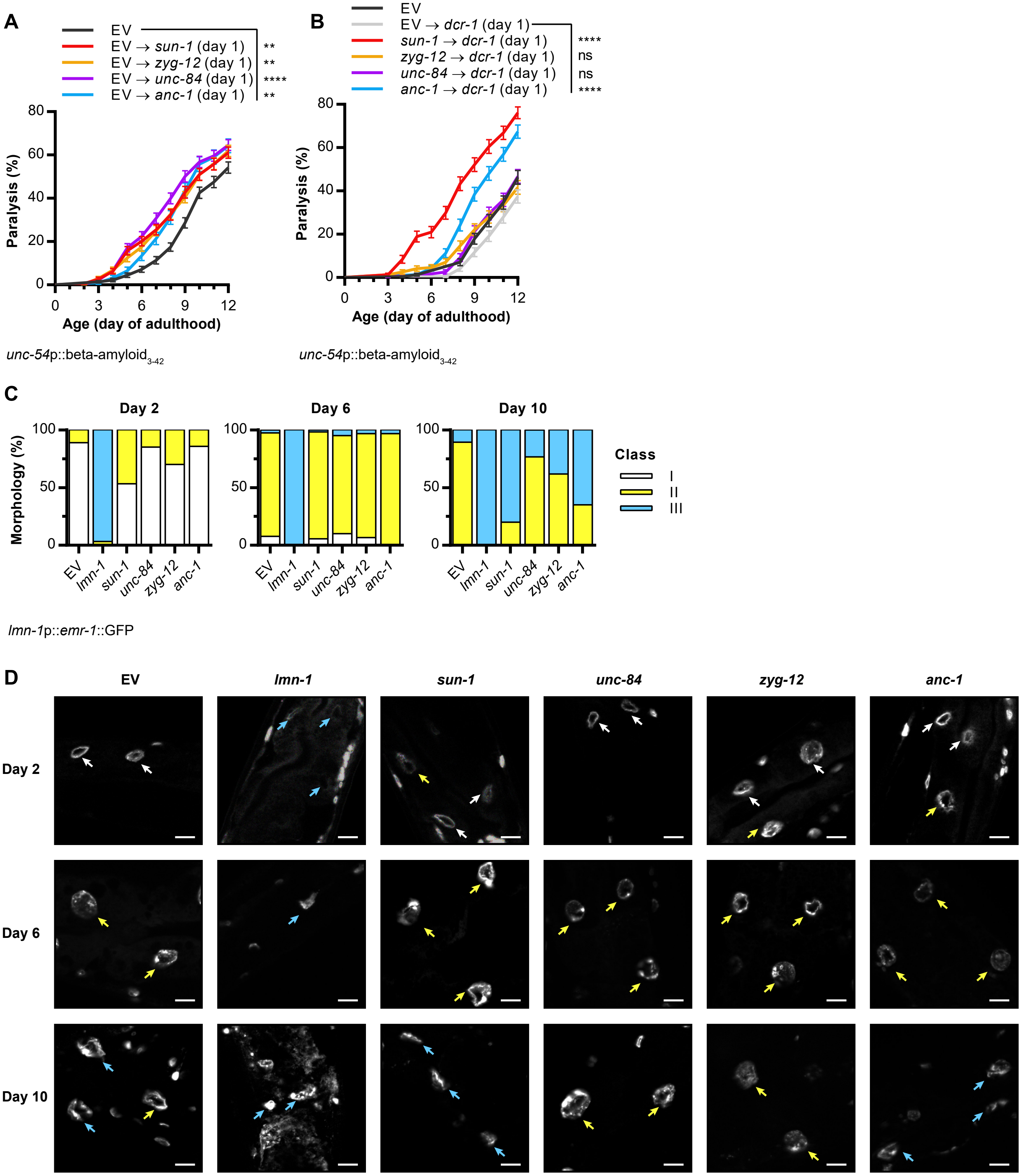
A
EV
EV → sun-1 (day 1) **
EV → zyg-12 (day 1) **
EV → unc-84 (day 1) ****
EV → anc-1 (day 1) **
Paralysis (%)
Age (day of adulthood)
unc-54p::beta-amyloid3-42
B
EV
EV → dcr-1 (day 1)
sun-1 → dcr-1 (day 1) ****
zyg-12 → dcr-1 (day 1) ns
unc-84 → dcr-1 (day 1) ns
anc-1 → dcr-1 (day 1) ****
Paralysis (%)
Age (day of adulthood)
unc-54p::beta-amyloid3-42
C
Day 2
Day 6
Day 10
Morphology (%)
EV
lmn-1
sun-1
unc-84
zyg-12
anc-1
Class
I
II
III
lmn-1p::emr-1::GFP
D
EV
lmn-1
sun-1
unc-84
zyg-12
anc-1
Day 2
Day 6
Day 10

Figure S1. Related to Figure 1. LINC proteins are required to protect against proteotoxicity in adulthood.

(A) Paralysis of animals that express A$\beta_{3\text{-}42}$ in their body-wall muscles (strain CL2006). The worms were treated with RNAi from day 1 of adulthood targeting one of the LINC components genes or fed with control bacteria that harbor the empty RNAi vector (EV). *sun-1,* n=337; *unc-84,* n=361; *zyg-12,* n=336; *anc-1,* n=215; EV, n=360. Animals were counted daily. The displayed results represent three independent repeats, except for *anc-1,* in which animals were counted in two independent repeats. Statistical significance was determined by the logrank test with a Bonferroni correction. ** *p*-value < 0.01, **** *p*-value < 0.0001. Error bars indicate SEM.

(B) Paralysis of animals that express A$\beta_{3\text{-}42}$ in their body-wall muscles (strain CL2006). The worms were treated from hatching with RNAi towards one of the genes that encode LINC components or fed with control bacteria (EV). On day 1 of adulthood, the worms were transferred onto *dcr-1* RNAi, to restore the expression of LINC components during adulthood. n=238-240 per condition, from two independent repeats. Statistical significance was determined by the logrank test with a Bonferroni correction. **** *p*-value < 0.0001; ns, not significant. Error bars indicate SEM.

(C, D) Morphologies of anterior intestinal cell nuclei (C), and their representative images (D), in *lmn-1*p::*emr-1*::GFP expressing worms (strain YG002). The worms were treated with RNAi towards the indicated LINC components. *lmn-1* RNAi serves as a positive control for defective nuclear morphology. The morphologies were categorized into three classes, as detailed in the main text. Briefly, the nuclear morphology classes are evenly distributed GFP (class I, white arrows), GFP puncta and/or convoluted nuclear

shape (class II, yellow arrows), and abnormal nuclear shapes (class III, blue arrows). For each condition, n=60 intestinal nuclei were counted from the anterior intestines of ten worms. Scale bar, 10 µm.

Figure S2

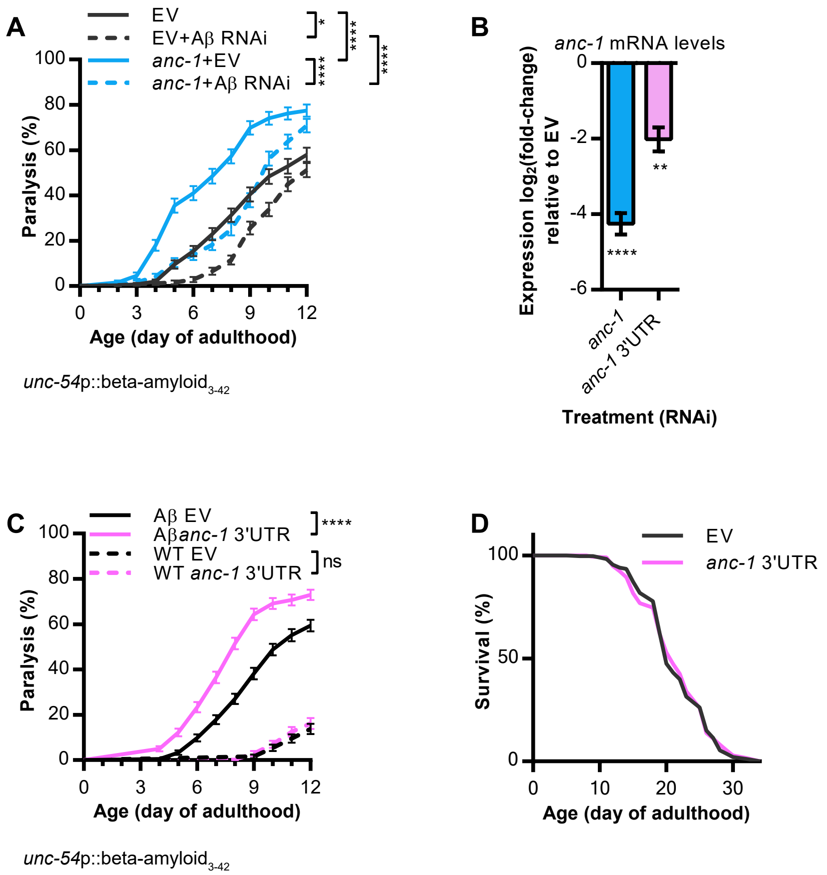
A
EV
EV+Aβ RNAi
anc-1+EV
anc-1+Aβ RNAi
*
****
****
****
Paralysis (%)
Age (day of adulthood)
unc-54p::beta-amyloid3-42
B
anc-1 mRNA levels
Expression log2(fold-change) relative to EV
****
**
anc-1
anc-1 3'UTR
Treatment (RNAi)
C
Aβ EV
Aβanc-1 3'UTR
WT EV
WT anc-1 3'UTR
****
ns
Paralysis (%)
Age (day of adulthood)
unc-54p::beta-amyloid3-42
D
EV
anc-1 3'UTR
Survival (%)
Age (day of adulthood)

Figure S2. Related to Figure 1. Relative knockdown efficiency and functional competence of RNAi constructs targeting *anc-1*.

(A) Mean paralysis of animals that express $A\beta_{3-42}$ in their body-wall muscles (Aβ worms, strain CL2006). The worms were treated from hatching with mixes of RNAi targeting *anc-1*, Aβ, and control bacteria that harbor the empty RNAi vector (EV). EV, n=240; EV+Aβ, n=242; *anc-1*+EV, n=239; *anc-1*+Aβ, n=240. Animals were counted daily in two independent experiments. Statistical significance was determined by the logrank test with a Bonferroni correction. * p-value < 0.05, **** p-value < 0.0001. Error bars indicate SEM.

(B) Quantitative real-time PCR (qPCR) measuring *anc-1* transcript levels in day 1 adult CF512 animals that were treated with one of two RNAi constructs that target *anc-1*; One from the *C. elegans* ORF-RNAi feeding library (Vidal) and the second is a construct we generated against the 3'UTR region. Statistical significance was determined by one-way ANOVA with Dunnett's multiple comparisons test. ** p-value < 0.01, **** p-value < 0.0001. Error bars indicate SEM.

(C) Mean paralysis of wild type animals (WT, strain N2), and Aβ worms (strain CL2006). The worms were treated from hatching with RNAi targeting the 3'UTR of *anc-1* (WT, n=241; Aβ, n=359), or fed with EV bacteria (EV: WT, n=240; Aβ, n=359). The animals were counted daily. The displayed results represent two independent repeats for WT worms, and three for Aβ animals. Statistical significance was determined by the logrank test with a Bonferroni correction. **** p-value < 0.0001; ns, not significant. Error bars indicate SEM.

(D) Survival curves of temperature sensitive sterile animals (strain CF512). The worms were treated from hatching with RNAi towards the 3’UTR of *anc-1* or fed with EV bacteria. *anc-1* 3’UTR, n=344; EV, n=360. Animals were counted daily. The displayed results represent three independent repeats. Statistical significance was determined by the logrank test.

Figure S3

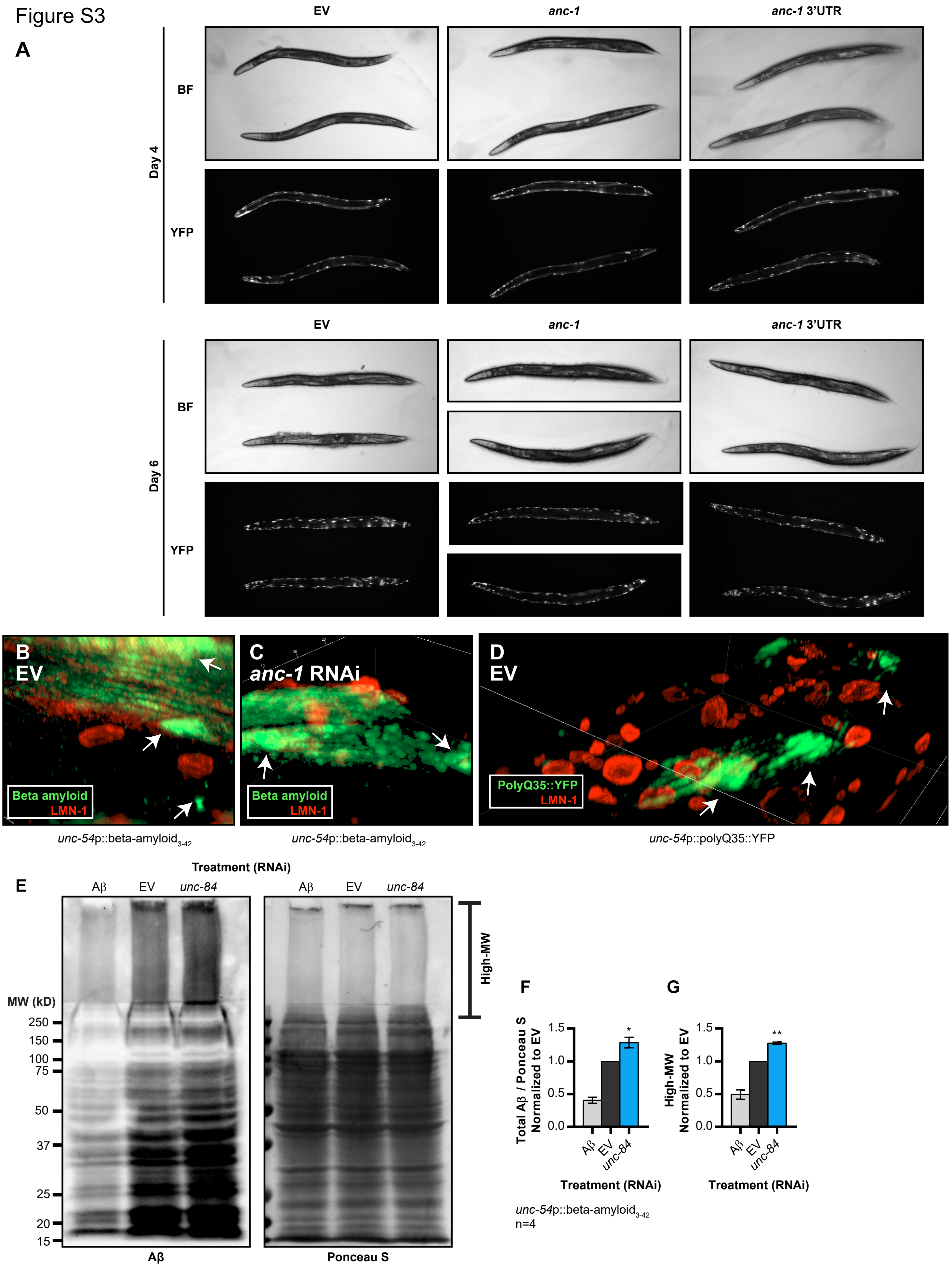
A
EV
anc-1
anc-1 3'UTR
Day 4
BF
YFP
Day 6
B
EV
Beta amyloid
LMN-1
unc-54p::beta-amyloid3-42
C
anc-1 RNAi
Beta amyloid
LMN-1
unc-54p::beta-amyloid3-42
D
EV
PolyQ35::YFP
LMN-1
unc-54p::polyQ35::YFP
E
Treatment (RNAi)
Aβ
EV
unc-84
MW (kD)
250
150
100
75
50
37
25
20
15
High-MW
Aβ
Ponceau S
F
Total Aβ / Ponceau S Normalized to EV
1.5
1.0
0.5
0.0
*
Treatment (RNAi)
G
High-MW Normalized to EV
**
Treatment (RNAi)
unc-54p::beta-amyloid3-42
n=4

Figure S3. Related to Figure 2. Hyper-aggregation of Aβ and polyQ35-YFP in untreated and *anc-1* RNAi-treated worms.

(A) Representative images of AM140 worms that express polyQ35-YFP in body-wall muscles, used to determine the number of foci and their fluorescence intensity. BF, brightfield.

(B, C) 3D reconstruction of Z-stack fluorescent images taken in the mid-body region of Aβ worms. Nuclei were labelled using an anti-LMN-1 antibody and Aβ was labelled using an anti-Aβ antibody (6E10). The results show that most Aβ foci do not overlap nuclei, and are not in their vicinity, in EV (B) and *anc-1* RNAi treated worm (C). White arrows point to Aβ accumulations.

(D) 3D reconstruction of Z-stack fluorescent images taken in the anterior intestinal region of polyQ35-YFP worms, with nuclei labelled using an anti-LMN-1 antibody, shows that polyQ35-YFP accumulations are not in the vicinity of nuclei, in untreated worms. White arrows point to polyQ35-YFP accumulations.

(E-G) Western blots of Aβ species in homogenates of day 3 adult Aβ worms that were either left untreated or exposed to Aβ or *unc-84* RNAi (E, representative). Mean total Aβ signals (F) and mean high-MW Aβ aggregates (G). F and G represent four independent experiments. The results show that unlike *anc-1* RNAi, *unc-84* knockdown does not reduce Aβ aggregation and modifies total Aβ levels. * p-value < 0.05, ** p-value < 0.01; ns, not significant. Error bars indicate SEM. Anti-Aβ antibody, 6E10.

Figure S4

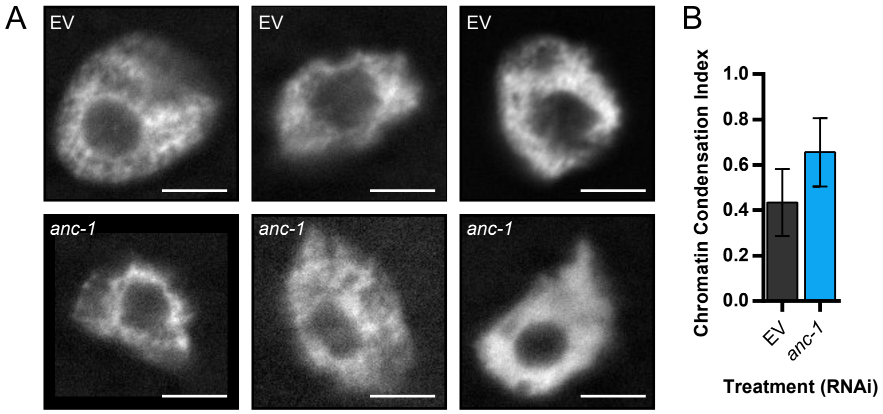
A
EV
EV
EV
anc-1
anc-1
anc-1
B
Chromatin Condensation Index
1.0
0.8
0.6
0.4
0.2
0.0
EV
anc-1
Treatment (RNAi)

Figure S4. Related to Figure 3. Chromatin condensation.

(A) DAPI staining of anterior intestinal nuclei of three separate day 6 adult worms (strain YG002), per condition. Scale bar, 5 µm.

(B) Chromatin condensation, as computationally measured in DAPI stained anterior intestinal nuclei of day 6 adult worms (strain YG002). EV, n=13; *anc-1*, n=19, nuclei were imaged in six separate worms per condition. Error bars indicate SEM.

Figure S5

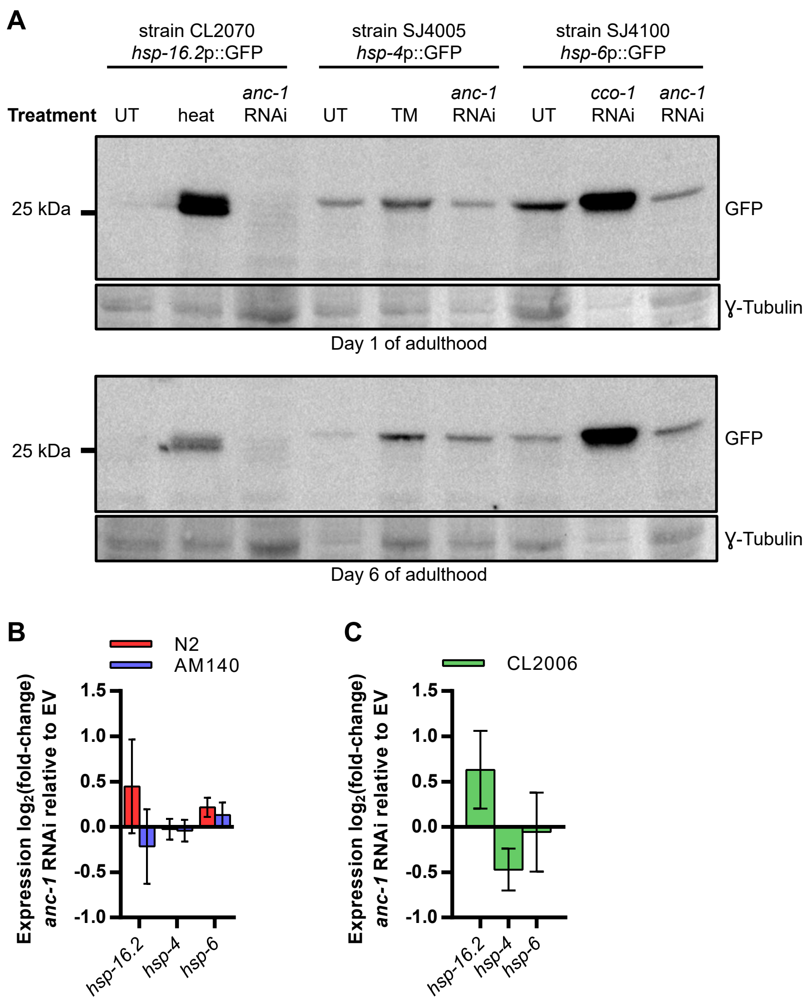

A
strain CL2070
hsp-16.2p::GFP
strain SJ4005
hsp-4p::GFP
strain SJ4100
hsp-6p::GFP
Treatment
UT
heat
anc-1 RNAi
UT
TM
anc-1 RNAi
UT
cco-1 RNAi
anc-1 RNAi
25 kDa
GFP
γ-Tubulin
Day 1 of adulthood
25 kDa
GFP
γ-Tubulin
Day 6 of adulthood
B
N2
AM140
Expression log2(fold-change) anc-1 RNAi relative to EV
1.5
1.0
0.5
0.0
-0.5
-1.0
hsp-16.2
hsp-4
hsp-6
C
CL2006
Expression log2(fold-change) anc-1 RNAi relative to EV
1.5
1.0
0.5
0.0
-0.5
-1.0
hsp-16.2
hsp-4
hsp-6

Figure S5. Related to Figure 3. ***anc-1*** **RNAi does not activate the heat shock response (HSR) and the unfolded protein response of the ER (UPR^{ER}) and of the mitochondria (UPR^{mt}).**

(A) Western blot analysis of days 1 and 6 adult worms that express GFP under promoters that are activated by the HSR, the UPR^{ER}, or the UPR^{mt}, using an anti-GFP (D5.1) antibody. The results show that *anc-1* knockdown does not induce any of these stress response mechanisms. Heat serves as a positive control for HSR induction. Tunicamycin (TM) serves as a positive control for UPR^{ER} induction. *cco-1* RNAi serves as a positive control for UPR^{mt} induction UT, untreated / EV.

(B, C) Expression fold-change of reporter genes in wild type and polyQ35-YFP day 6 old animals, as measured by RNA-Seq (B), and in Aβ worms, as measured by quantitative real-time PCR (qPCR) (C). Three independent repeats show that *anc-1* knockdown does not modulate the expression levels of HSR, UPR^{ER}, and UPR^{mt} target genes in the presence and absence of proteotoxic stress.

Figure S6

Gene Ontology (GO) Biological Process (BP) Gene Set Enrichment Analysis (GSEA)

Wild-type (strain N2)

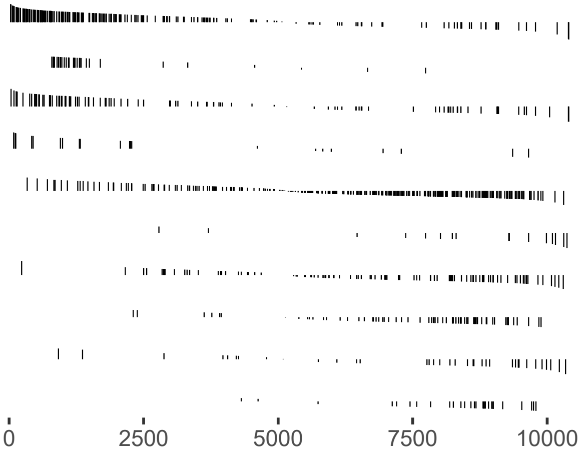

Pathway	*NES*	*pval*	*padj*
**GO:0045087 innate immune response**	2.27	1.1e−04	1.6e−02
**GO:0002181 cytoplasmic translation**	2.26	1.4e−04	1.6e−02
GO:0050829 defense response to Gram−negative bacterium	1.69	4.7e−04	1.8e−02
GO:0042338 cuticle development involved in collagen and cuticulin−based cuticle molting cycle	1.75	2.2e−03	4.9e−02
**GO:0009792 embryo development ending in birth or egg hatching**	−1.44	2.2e−03	4.9e−02
**GO:0061158 3'−UTR−mediated mRNA destabilization**	−1.93	1.5e−03	4.2e−02
**GO:0000003 reproduction**	−1.78	6.4e−04	2.0e−02
**GO:0000398 mRNA splicing, via spliceosome**	−2.10	4.8e−04	1.8e−02
**GO:0045892 negative regulation of transcription, DNA−templated**	−1.94	3.6e−04	1.8e−02
**GO:0040027 negative regulation of vulval development**	−2.17	3.5e−04	1.8e−02

PolyQ35-YFP (strain AM140)

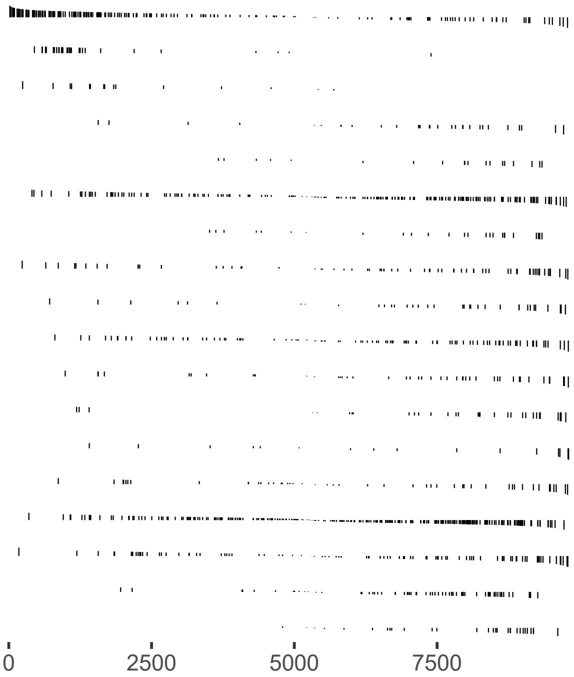

Pathway	*NES*	*pval*	*padj*
**GO:0045087 innate immune response**	2.22	1.6e−04	1.0e−02
**GO:0002181 cytoplasmic translation**	2.34	1.8e−04	1.0e−02
GO:0019722 calcium−mediated signaling	1.83	1.8e−03	2.4e−02
GO:0040028 regulation of vulval development	−1.76	2.7e−03	3.2e−02
GO:0000422 autophagy of mitochondrion	−1.83	2.3e−03	2.8e−02
GO:0002119 nematode larval development	−1.50	1.6e−03	2.3e−02
GO:0000045 autophagosome assembly	−1.89	1.4e−03	2.0e−02
GO:0030154 cell differentiation	−1.77	9.5e−04	1.5e−02
**GO:0045892 negative regulation of transcription, DNA−templated**	−1.84	9.3e−04	1.5e−02
**GO:0000003 reproduction**	−1.68	7.5e−04	1.4e−02
GO:0001708 cell fate specification	−1.86	7.0e−04	1.4e−02
GO:0001764 neuron migration	−1.95	6.9e−04	1.4e−02
**GO:0061158 3'−UTR−mediated mRNA destabilization**	−1.93	6.7e−04	1.4e−02
GO:0006355 regulation of transcription, DNA−templated	−1.91	4.7e−04	1.4e−02
**GO:0009792 embryo development ending in birth or egg hatching**	−1.72	2.9e−04	1.0e−02
GO:0006357 regulation of transcription by RNA polymerase II	−1.90	2.5e−04	1.0e−02
**GO:0000398 mRNA splicing, via spliceosome**	−2.11	2.4e−04	1.0e−02
**GO:0040027 negative regulation of vulval development**	−2.21	2.3e−04	1.0e−02

Processes that are enriched in both strains
Processes associated to proteostasis
Processes associated to transcription

Figure S6. Related to Figure 3. Gene Ontology (GO) Gene Set Enrichment Analysis (GSEA).

Enrichment (upregulation, NES > 0) or Depletion (downregulation, NES < 0) of GO biological processes based on the differential gene expression measured in wild type (strain N2) and polyQ35-YFP (strain AM140) animals treated with *anc-1* RNAi. Adjusted *p*-value threshold set at 0.05. "*Pathway*" is the GO annotation gene-set; "*Gene ranks*" are the positions of the genes that make up the corresponding *Pathway* relative to all the genes measured by RNA-seq, which are sorted by their $\log_2$(fold-change) from highest to lowest. "*NES*" are normalized enrichment scores; "*pval*" is the *p*-value prior to multiple comparisons correction; "*padj*" is the FDR-adjusted *p*-values.

Figure S7

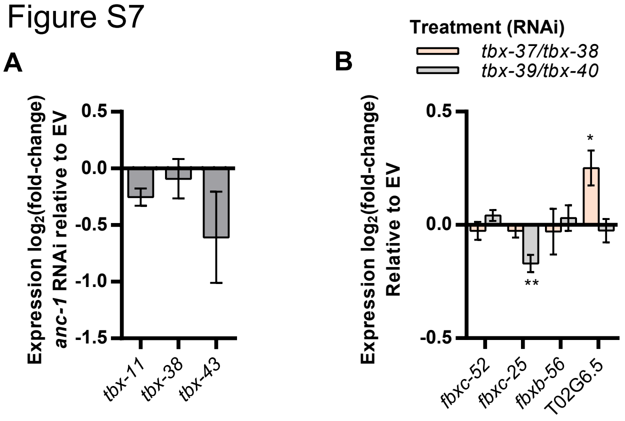

*unc-54*p::beta-amyloid$_{3-42}$
n=3

*unc-54*p::polyQ35::YFP
n=3

Figure S7. Related to Figure 4. ANC-1 modulates the expression of T-box transcription factors and components of the SCF complex.

(A) Expression fold-change of T-box transcription factor-coding genes in day 6 adult Aβ worms, as measured by qPCR, shows that knocking down *anc-1* tends to reduce the expression of T-box transcription factors in these animals. Three independent repeats.

(B) Expression fold-change of F-box protein coding genes in day 6 adult polyQ-35-YFP worms, as measured by qPCR. Three independent repeats indicate that the knockdown of *tbx-37*/*tbx-38* modulates the expression of *T02G6.5* and that the knockdown of *tbx-39*/*tbx-40* modulates the expression of *fbxc-25.*

* $p < 0.05$, ** $p < 0.01$. Error bars indicate SEM.

Figure S8

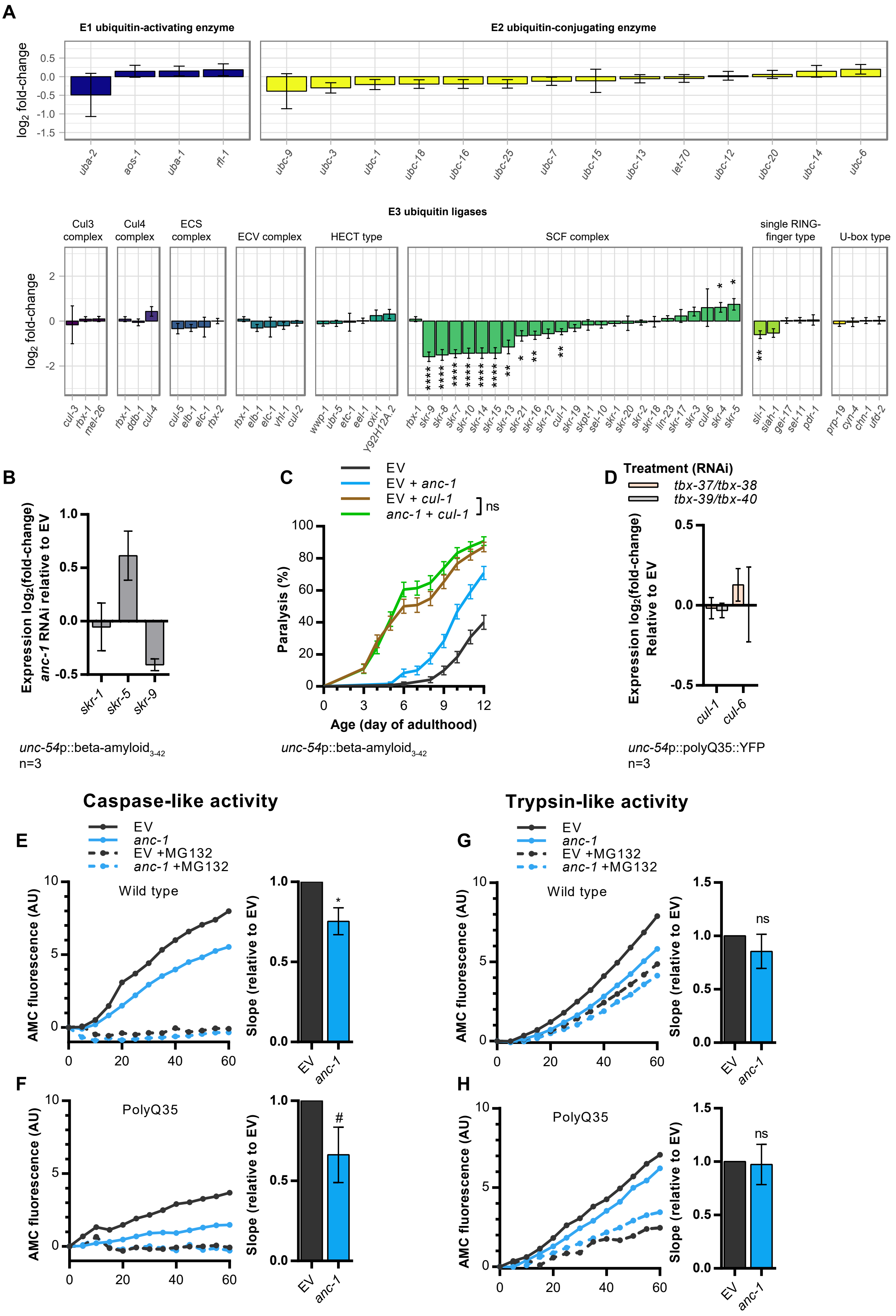

A
E1 ubiquitin-activating enzyme
E2 ubiquitin-conjugating enzyme
log2 fold-change
uba-2
aos-1
uba-1
rfl-1
ubc-9
ubc-3
ubc-1
ubc-18
ubc-16
ubc-25
ubc-7
ubc-15
ubc-13
let-70
ubc-12
ubc-20
ubc-14
ubc-6
E3 ubiquitin ligases
Cul3 complex
Cul4 complex
ECS complex
ECV complex
HECT type
SCF complex
single RING-finger type
U-box type
B
Expression log2(fold-change) anc-1 RNAi relative to EV
skr-1
skr-5
skr-9
unc-54p::beta-amyloid3-42
n=3
C
EV
EV + anc-1
EV + cul-1
anc-1 + cul-1
ns
Paralysis (%)
Age (day of adulthood)
unc-54p::beta-amyloid3-42
D
Treatment (RNAi)
tbx-37/tbx-38
tbx-39/tbx-40
Expression log2(fold-change) Relative to EV
cul-1
cul-6
unc-54p::polyQ35::YFP
n=3
Caspase-like activity
Trypsin-like activity
E
F
G
H
EV
anc-1
EV +MG132
anc-1 +MG132
Wild type
PolyQ35
AMC fluorescence (AU)
Slope (relative to EV)

Figure S8. Related to Figure 5. ANC-1 modulates the expression of SCF complex E3 ubiquitin ligases and proteasome-mediated degradation.

(A) Expression fold-change of ubiquitin modifying enzymes coding genes when *anc-1* is knocked down in polyQ35-YFP (strain AM140) animals, as measured by RNA-seq. The gene list was obtained from the 'Ubiquitin mediated proteolysis in *C. elegans*' entry in the KEGG pathway database. Statistical significance was determined by T-test with BH Procedure.

(B) Expression fold-change of genes that code Skp1 homologs in day 6 adult Aβ worms, as measured by qPCR, shows that *anc-1* knockdown regulates the expression of SCF complex ubiquitin ligase components. Three independent repeats.

(C) Mean paralysis of Aβ worms that were treated from hatching with mixes of RNAi towards *anc-1*, *cul-1*, and EV bacteria. n=119-124 per condition from one experiment shows that *anc-1* RNAi leads to a small non-significant increase in Aβ proteotoxicity when *cul-1* is knocked down. Statistical significance was determined by the logrank test with a Bonferroni correction. Error bars indicate SEM.

(D) Expression fold-change of Cullin coding genes in day 6 adult polyQ35-YFP worms, as measured by qPCR. Three independent repeats show that the expression of *cul-1* and *cul-6* is not regulated by T-box transcription factors.

(E, F) *In vitro* caspase-like proteasome activity of day 6 adult wild type (E) and polyQ35-YFP (F) worm homogenates, and the mean slopes of three independent repeats, show that the knockdown of *anc-1* impairs this activity.

(G, H) *In vitro* trypsin -like proteasome activity of day 6 adult wild type (G) and polyQ35-YFP (H) worm homogenates, and the mean slopes of three independent repeats,

show no significant difference between untreated worms and their counterparts that were fed with *anc-1* RNAi bacteria.

[#] $p < 0.1$, * $p < 0.05$, ** $p < 0.01$, *** $p < 0.001$, **** $p < 0.0001$; ns, not significant. Error bars indicate SEM.

Supplemental Tables

Table S1. Related to Figure 3. Enrichment of transcription factor binding motifs that are regulated by *anc-1* RNAi in both wild type and polyQ35-YFP animals.

**Rank**[a]	**Gene ID**	**Raw score**[b]	***p*-value**[c]
1	*tbx-38*	1.253635	**8.60E-05**
2	B0310.2	1.270693	**0.005305**
3	*tbx-33*	1.25718	**0.020352**
4	*tbx-43*	1.306603	0.054775
5	F19F10.1	1.104687	0.253082
6	*ztf-3*	2.731587	0.524245
7	*sex-1*	1.129033	0.530266
8	*ztf-16*	1.055888	0.663082
9	*ceh-37*	0.508321	0.740343
10	*dpy-27*	0.119191	0.830897
11	*xbp-1*	0.950913	0.946353
12	*ztf-11*	1.558917	0.999194
13	*lsy-27*	1.049056	0.999951
14	*die-1*	1.397303	0.999996

[a] Rank sorted by *p*-value.
[b] Score output of the enrichment analysis performed using the R package ‘PWMEnrich’.
[c] Values in bold indicate a *p*-value < 0.05.

Table S2. Related to experimental procedures. Primer sequences.

Primer name	Primer sequence
anc-1 3'UTR - RNAi - forward	TCGAGATGCTAGCTTGTTTGGTTCCACATTGC
anc-1 3'UTR - RNAi - reverse	TCGAGATCTCGAGAATCAACTGGGATGTATGGG
anc-1-qRTPCR - forward	TGAGATAACAGCCGAAGTTG
anc-1-qRTPCR - reverse	TGAGCAATAGCATCGTTGAG
tbx-11-qRTPCR - forward	GTTCTCACCATCTACGAAGG
tbx-11-qRTPCR - reverse	CGAGATCCAGTTGAATACGG
tbx-38-qRTPCR - forward	TTTTAGTGGGGAGCAGTTCC
tbx-38-qRTPCR - reverse	TTGCCATTCGAACGAAATCC
tbx-43-qRTPCR - forward	GATCGGCAAATTTCAGTTCC
tbx-43-qRTPCR - reverse	TTGATGCTCTCACGAAATCC
skr-1-qRTPCR - forward	AAAGAACTGACGACATAGGC
skr-1-qRTPCR - reverse	CAAGAAGTCCCTTGATGTCC
skr-5-qRTPCR - forward	AATTGCCAATCAATCCAAGC
skr-5-qRTPCR - reverse	GGTTTGGGGATATCTTCAGC
skr-9-qRTPCR - forward	CCAAAGCATGTCAATGTTCC
skr-9-qRTPCR - reverse	GGCAACAATCTTACATCCG
fbxc-52-qRTPCR - forward	AAATGAACTTGGATGTTGGG
fbxc-52-qRTPCR – reverse	TTGTCGATTTCTTTCGTTCC
fbxc-25-qRTPCR – forward	CATTGGATGATGAATCTCAAGC
fbxc-25-qRTPCR – reverse	GAAAGCCTCTGTAGAGTTGC
fbxb-56-qRTPCR – forward	TTTCAACTCATGTCACATTCG
fbxb-56-qRTPCR – reverse	ATTTCACAAGGCGTTTAGC
T02G6.5-qRTPCR – forward	GAGAAGAATCCGAGTCAACG
T02G6.5-qRTPCR – reverse	CAATCCAGTTGTTCGAGTCC
cul-1-qRTPCR – forward	AAAATGTTGAGGAGGATCGG
cul-1-qRTPCR – reverse	TCAACCTTTGGCTTGAATCG
cul-6-qRTPCR - forward	CGAGTGGATTTGACAACGG
cul-6-qRTPCR - reverse	GTGTGGGTGAGACTTTTCC

cdc-42-qRTPCR - forward	GATGTTGGAGAGAAGTTGGC
cdc-42-qRTPCR - reverse	CTTCTTCTCCTGTTGTGGTGG
pmp-3-qRTPCR - forward	GCTAACTGAATGGAGAATGG
pmp-3-qRTPCR - reverse	AGGTACAGGTAGAAGAATGC
Y45F10D.4-qRTPCR - forward	GTCTTCCACCAGTCAAACTTC
Y45F10D.4-qRTPCR - reverse	GCCTTCTTAGTCTGCTTCTTCTG

Supplemental Movies

Movie S1.

3D reconstruction of Z-stack fluorescence images taken in the vulva region of Aβ worms (strain CL2006), with nuclei labelled by an anti-LMN-1 antibody and Aβ labelled using an anti-Aβ antibody, shows that Aβ accumulations mostly do not overlap with the nuclei, and are not in their vicinity. Green, Aβ. Red, LMN-1. Video format AVI (MJPEG).

Movie S2.

3D reconstruction of Z-stack fluorescence images taken in the anterior intestinal region of polyQ35-YFP worms (strain AM140), with nuclei labelled by an anti-LMN-1 antibody, shows that polyQ35-YFP accumulations are not in the vicinity of nuclei. Green, polyQ35-YFP. Red, LMN-1. Video format AVI (MJPEG).

Movie S3.

3D reconstruction of an anterior intestinal nucleus stained with DAPI, in day 6 adult worms (strain YG002), treated with control (EV). Video format AVI (MJPEG).

Movie S4.

3D reconstruction of an anterior intestinal nucleus stained with DAPI, in day 6 adult worms (strain YG002), treated with anc-1 RNAi. Video format AVI (MJPEG).

Experimental Procedures

Caenorhabditis elegans

Standard methods were used for the maintenance and manipulation of *C. elegans* (Stiernagle, 2006). For the experiments conducted in this study, hermaphrodite *C. elegans* were synchronized using hypochlorite and potassium hydroxide, and cultured at 20 °C, unless stated otherwise. Strains used in this study: Wild type (N2, Bristol, RRID:WB-STRAIN:N2_(ancestral)), CL2006 (pCL12[*unc-54*p::human A$\beta_{3\text{-}42}$], pRF4(*rol-6*(*sulO06*)), RRID:WB-STRAIN:CL2006), CL2070 (dvIs70[*hsp-16.2*p::GFP + *rol-6*(su1006)], RRID:WB-STRAIN:CL2070), SJ4005 (zcIs4 [*hsp-4*::GFP] V, RRID:WB-STRAIN:SJ4005), SJ4100 (zcIs13[*hsp-6*::GFP] V, RRID:WB-STRAIN:SJ4100), were provided by the CGC (Caenorhabditis Genetics Center, Minneapolis, MN), which is funded by NIH Office of Research Infrastructure Programs (P40 OD010440). YG002 (*lmn-1*p::*emr-1*::GFP) was generously provided by Prof. Yosef Gruenbaum (the Hebrew University of Jerusalem, Israel). AM140 (rmIs132[*unc-54*p::Q35::YFP], RRID:WB-STRAIN:AM140) and AM101 (rmIs110[F25B3.3p::Q40::YFP], RRID:WB-STRAIN:AM101) were generously provided by Dr. Richard I. Morimoto (Northwestern University, IL, USA). CF512 (*fer-15*(*b26*), *fem-1*(*hc17*), RRID:WB-STRAIN:CF512) was generously provided by Dr. Andrew Dillin (Berkeley, CA, USA). All strains were routinely maintained at 15 °C, and all experiments were performed at 20 °C, unless stated otherwise. Worms were fed with the *Escherichia coli* strain HT115 (WB Cat# HT115(DE3), RRID:WB-STRAIN:HT115(DE3)) that expresses dsRNA for RNA interference. Bacteria were

grown overnight at 37 °C in LB media supplemented with 100 µg/ml ampicillin (Formedium, 69-52-3).

RNA interference (RNAi)

NG plates supplemented with 100 µg/ml ampicillin were seeded with bacterial cultures. To induce RNAi, the seeded plates were treated with 120 µl 100 mM Isopropyl-β-D-Thiogalactopyranoside (IPTG) (Inalco, 1758-1400). For RNAi of two genes simultaneously, equal volumes of two bacterial cultures were mixed, and seeded on plates. The bacteria were obtained from the *C. elegans* ORF-RNAi feeding library (Vidal), unless stated otherwise. The sequence specificity of all the RNAi constructs was verified by sequencing. The *anc-1* 3'UTR RNAi construct was generated by PCR amplifying part of the 3'UTR-region of the *anc-1* transcript using Paq5000™ DNA Polymerase (Agilent Technologies, 600682). The primer sequences are detailed in Table S2. The PCR product was purified using a PureLink® PCR Purification Kit (Invitrogen, K3100-01), digested by NheI-HF and XhoI restriction enzymes (New England Biolabs, R3131, R0146), and cloned into the pL4440 plasmid using the T4 DNA Ligase (New England Biolabs, M0202). The cloned plasmid was transformed to *E. coli* DH5α to increase plasmid quantity, and then transformed to *E. coli* HT115 to be used as a food source for RNAi knockdown. The cloning and transformation were validated by Standard-Seq (Macrogen Inc.) using the M13F primer. The Aβ RNAi targets the sequence within the *Homo sapiens* gene APP that encodes Aβ and that is expressed in the *C. elegans* strain CL2006:

GATGCAGAATTCCGACATGACTCAGGATATGAAGTTCATCATCAAAAATTGGTGTTCTT

TGCAGAAGATGTGGGTTCAAACAAAGGTGCAATCATTGGACTCATGGTGGGCGGTGTTG
TCATAGCG

Paralysis assay

Synchronized CL2006 eggs were placed on NG-ampicillin plates seeded with HT115 bacteria and supplemented with IPTG. On the first day of adulthood, 120 randomly picked animals per treatment were transferred onto 60 mm NG-ampicillin plates seeded with bacteria, 24 animals per plate. The worms were assessed daily for paralysis by gently tapping their noses with a platinum wire. If worms did not move, they were further incentivized by placing a hot platinum wire in their proximity. Living worms that failed to move their central body regions were scored as paralyzed and removed from the plates. To avoid scoring old animals as paralyzed, paralysis assays were terminated at day 12 of adulthood.

Nuclear morphology and chromatin condensation

For each treatment, synchronized YG002 eggs were placed on NG-ampicillin plates seeded with HT115 bacteria and supplemented with IPTG. Starting at day 1 of adulthood, 50 worms per condition were picked daily to avoid progeny. On the indicated days, the worms were washed twice with M9 to remove bacteria followed by two washes with water, and incubated on ice for ten minutes. Samples were fixed with 4% paraformaldehyde in MRWB buffer (80 mM KCl, 20 mM NaCl, 10 mM Na_2EGTA, 5 mM spermidine, 25% methanol). The worms were frozen in -80C, thawed, and incubated for 30 minutes on ice with occasional agitation. The worms were washed three times in PBS, and 10-20 animals were mounted per condition in 5% (w/v) n-propyl gallate (Sigma-Aldrich, P3130), 100 mM Tris-HCl pH 9.0, 70% (v/v) glycerol, and 20 µg/ml 4',6-Diamidino-2-phenylindole (DAPI), for fluorescence microscopy imaging. DIC and

fluorescence images were acquired using a Zeiss Axio Observer.Z1 inverted microscope equipped with an AxioCam HRm Zeiss camera, with optical sectioning using using Zeiss ApoTome.2, Plan-Apochromat 63x/1.40 oil immersion objective, and the Zeiss ZEN 2 (blue edition) software. For nuclear morphology, we imaged the anterior intestines across the worm diameter, in 2 μm slices. The nuclei were categorized into classes, based on nuclear features that were set by Haithcock and colleagues, as detailed in the main text (Haithcock et al., 2005). For nuclear morphology, we imaged anterior intestinal nuclei in 200 nm slices. Chromatin condensation index was calculated using a MATLAB tool designed to measure chromatin *in vivo*, developed by Sosnik and colleagues (Sosnik, Vieira, Webster, Siegfried, & McCusker, 2017). In brief, Chromatin condensation index is defined as the number of edges detected using the Sobel edge detection method within a nuclear cross-section relative to the nuclear area. For more details, see work by Irianto and colleagues (Irianto, Lee, & Knight, 2014).

Lifespan assay

Synchronized CF512 eggs were placed on NG-ampicillin plates seeded with HT115 bacteria and supplemented with IPTG. CF512 animals are heat-sensitive sterile; to avoid progeny, the eggs were placed at 25 °C. On the first day of adulthood the worms were moved to 20 °C, and 120 randomly picked animals per treatment were transferred onto 60 mm NG-ampicillin plates seeded with bacteria, 12 animals per plate. Worms that failed to move when tapped twice gently with a platinum wire and placing a hot platinum wire in their proximity were scored as dead. Survival rates were recorded every 2-3 days until the last surviving worm perished.

Thrashing assay

Synchronized eggs were placed on NG-ampicillin plates seeded with HT115 bacteria and supplemented with IPTG. On each time-point, 20 randomly picked animals were sequentially placed in 5 µl M9 buffer drop on top of a microscope slide and allowed thirty seconds of recovery. Afterwards, the number of body bends of each worm was counted for the duration of thirty seconds. One body bend is the formation of an angle between the head and tail of the worm in a pre-selected lateral movement direction.

Native Agarose Gel Electrophoresis (NAGE)

We performed the assay as described by its developers (Holmberg & Nollen, 2013). For each time-point, 4,500 synchronized AM140 eggs were placed on NG-ampicillin plates seeded with HT115 bacteria and supplemented with IPTG. The worms were washed daily with M9 to discard of progeny. On each time-point, the worms were washed with M9 to remove bacteria, and resuspended them twice in PBS supplemented with Protease Inhibitor Cocktail Set III (Merck / Calbiochem, 539134) diluted 1:1000. Afterwards, the buffer volume was minimized, and the worms were flash frozen the worms in liquid nitrogen. After thawing the samples, a small amount of 0.5 mm zirconium oxide beads (Next Advance, ZrOB05) was added to the collected worms. The worms were homogenized at 4 °C using a Bullet Blender® (Next Advance) set to speed nine – three times for ten seconds each. Worm debris was removed by centrifuging at 780 x g for three minutes at 4 °C and transferring away the soluble upper post-debris fraction. The protein content of the post-debris fraction was quantified using the BCA Protein Assay Kit (Pierce™, 23227) according to its suggested protocol. For each sample, 100 µg were loaded onto a 1% agarose gel and ran on Owl™ EasyCast™ B1 Mini Gel Electrophoresis Systems (Thermo Scientific) at 4 °C, 40 V for 15 hours. The gels were

visualized using a Typhoon FLA 9500 (GE Healthcare) set to Alexa 488 Fluorescence, PMT 500, pixel size of 50 µm. The YFP signal intensities were quantified using Image Lab™ version 6.0.0 build 25 Standard Edition (Bio-Rad Laboratories). High molecular weight regions were defined, with the background signal subtracted from each region of interest. The signal intensities were normalized to the untreated samples.

Counting polyglutamine foci

Synchronized eggs were placed on NG-ampicillin plates seeded with HT115 bacteria and supplemented with IPTG. On each time-point, worms were picked onto NG-ampicillin free from bacteria, immobilized in 20 mM sodium azide diluted in M9 buffer, and their entire bodies were individually imaged in a Nikon AZ100 microscope equipped with a Nikon DS-Fi2 camera, using the software NIS-Elements BR (version 4.13). The fluorescent polyQ35-YFP foci were quantified using the software worMachine (Hakim et al., 2018).

SDS-PAGE and western blot

For each condition, 10,000 to 15,000 synchronized eggs were placed on NG-ampicillin plates seeded with HT115 bacteria and supplemented with IPTG. The worms were washed daily with M9 to discard of progeny. On the indicated ages, the worms were washed with M9 to remove bacteria, and resuspended twice in PBS supplemented with 1:1000 Protease Inhibitor Cocktail Set III (Merck / Calbiochem, 539134).

When testing the induction of the HSR, CL2070 worms were exposed heat at 33 °C for 3 h, and were then collected as described. When testing the induction of the UPR^{ER}, SJ4005 worms were exposed to 10 µg/ml Tunicamycin at 20 °C for 3 h, and were then collected as described. When testing the induction of the UPR^{mt}, SJ4100 worms

were treated with *cco-1* RNAi from hatching, and were collected as described. Afterwards, the buffer volume was minimized, and the worms were flash frozen in liquid nitrogen. After thawing the samples, a small amount of 0.5 mm zirconium oxide beads (Next Advance, ZrOB05) was added to the collected worms. The worms were homogenized at 4 °C using a Bullet Blender® (Next Advance) set to speed eight, for three minutes, and then set to speed nine and for an additional two minutes. The homogenates were centrifuged at 780 x g for three minutes at 4 °C and the worm debris was separated from the soluble upper post-debris fraction. The protein content of the worm debris (anti-Aβ western blot) and post-debris fraction (anti-ubiquitin, anti-polyQ, and anti-GFP western blot) were quantified using the BCA Protein Assay Kit (Pierce™, 23227) according to its suggested protocol. For each sample, 100 µg of protein were supplemented with a 4X sample loading buffer (40% glycerol, 0.2 M Tris-HCl pH 6.8, 0.5% Bromophenol blue, 4% SDS), 10.7% (v/v) 2-mercaptoethanol (Sigma, M3148), and incubated at 95 °C for ten minutes. The samples were loaded into wells of a Tris polyacrylamide gel (Stacking: 5% Acry/Bis (29:1), 0.125 M Tris pH 6.8, 0.1% SDS, 0.1% APS, 0.1% TEMED; Resolving: 10% Acry/Bis (29:1), 0.375 M Tris pH 8.8, 0.1% SDS, 0.04% APS, 0.1% TEMED). Gel electrophoresis took place in a Mini-PROTEAN® Tetra Cell (Bio-Rad Laboratories) and running buffer (0.19 M Glycine, 25 mM Tris, 3.5 mM SDS) at room temperature, 100 V for two hours. Each gel was loaded with a Precision Plus Protein™ Dual Color Standards as a protein marker (Bio-Rad, 1610374). Gel electrophoresis was halted when the Bromophenol blue dye reached the bottom of the gel. Afterwards, the proteins were transferred onto a methanol-activated 0.45 µm pore PVDF membrane (Millipore, IPVH00010) (In anti-Aβ western blot a 0.2 µm pore

nitrocellulose membrane (Pall Corporation, 66485) was used) using a Criterion™ Blotter (Bio-Rad Laboratories) at room temperature, 60 mAh overnight for 15-17 hours. Post-transfer, total protein content was stained using 0.1% (w/v) Ponceau S (Sigma-Aldrich, P3504) in 1% (v/v) acetic acid, and the membrane was visualized in a Molecular Imager® Gel Doc™ XR+ (Bio-Rad Laboratories). The membrane was cleared from Ponceau S by TBST (10 mM Tris pH 8.0, 0.15 M NaCl supplemented with 0.05% (v/v) Tween-20) and blocked in 5% (w/v) non-fat milk for one hour, then with the primary antibody for two hours, and finally with the secondary antibody. All incubations took place at room temperature. The primary antibodies used were 1:1,000 mono- and poly-ubiquitinated conjugates monoclonal antibody (FK2) (Enzo Life Sciences Cat# BML-PW8810, RRID:AB_10541840); 1:1,000 purified anti-β-Amyloid, 1-16 monoclonal antibody (6E10) (BioLegend Cat# 803001, RRID: AB_2564653); 1:2,000 mouse anti-polyglutamine-expansion (5TF1-1C2) (Millipore Cat# MAB1574, RRID:AB_94263); for blotting polyQ35-YFP (strain AM140), 1:2,000 mouse anti-GFP monoclonal antibody (11E5) (Thermo Fisher Scientific Cat# A-11121, RRID:AB_221567) was used; for blotting GFP under the promoters of heat-shock proteins (strains CL2070, SJ4005, SJ4100), 1:2,000 rabbit anti-GFP monoclonal antibody (D5.1) (Cell Signaling Technology Cat# 2956, RRID:AB_1196615) was used; mouse anti-gamma-tubulin monoclonal antibody, unconjugated, (GTU-88) (Sigma-Aldrich Cat# T6557, RRID:AB_477584). The secondary antibodies used were 1:10,000 Peroxidase AffiniPure Donkey Anti-Mouse IgG (H+L) (Jackson ImmunoResearch Labs Cat# 715-035-151, RRID:AB_2340771); Peroxidase AffiniPure Donkey Anti-Rabbit IgG (H+L) (Jackson ImmunoResearch Labs Cat# 711-035-152, RRID:AB_10015282). Chemiluminescence

was detected by exposing the antibody-bound membrane to 1.25 mM luminol, 0.2 mM p-coumaric acid, 0.0083% hydrogen peroxide, and 100 mM Tris pH 8.5, and using a Molecular Imager® Gel Doc™ XR+ (Bio-Rad Laboratories). The Ponceau S staining and chemiluminescence were quantified using Image Lab™, version 6.0.0 build 25, standard edition (Bio-Rad Laboratories). High molecular weight and low molecular weight regions were defined, with the background signal subtracted from each region of interest. The signal intensities were normalized to the untreated samples.

Immunofluorescence

For each treatment, synchronized eggs were placed on NG-ampicillin plates seeded with HT115 bacteria and supplemented with IPTG. Starting at day 1 of adulthood, 50 worms per condition were picked daily to avoid progeny. On the indicated days, the worms were washed twice with M9 to remove bacteria followed by two washes with water, and incubated on ice for ten minutes. Samples were fixed with 4% paraformaldehyde in MRWB buffer (80 mM KCl, 20 mM NaCl, 10 mM Na_2EGTA, 5 mM spermidine, 25% methanol). The worms were frozen in -80C, thawed, and incubated for 30 minutes on ice with occasional agitation. The worms were washed twice in Tris-Triton buffer (100 mM Tris-HCl pH 7.4, 1% Triton X-100, 1 mM EDTA), incubated for 2 h at 37 °C in Tris-Triton buffer supplemented with 1% β-Mercaptoethanol, with mild agitation, washed in BO_3 buffer (25 mM H_3BO_3, 12.5 mM NaOH), and incubated for 15 minutes in BO_3 buffer supplemented with 10 mM DTT, with mild agitation. The worms were then washed once in BO_3 buffer, and incubated in BO_3 buffer supplemented with 0.3% H_2O_2 for 15 minutes at room temperature, with mild agitation. After washing once with BO_3 buffer, and for 15 minutes at room temperature with washing buffer (1X PBS,

0.1% BSA, 0.5% Triton X-100, 1 mM EDTA), the worms were incubated for one hour at room temperature in blocking buffer (1X PBS, 1% BSA, 0.5% Triton X-100, 1 mM EDTA) and stained overnight at 4 °C with the primary antibody in blocking buffer. The worms were washed three times in washing buffer, and stained for two hours at room temperature with fluorophore conjugated secondary antibodies followed by ten washes in washing buffer. 10-20 animals were mounted per condition in 5% (w/v) n-propyl gallate (Sigma-Aldrich, P3130), 100 mM Tris-HCl pH 9.0, 70% (v/v) glycerol, and 20 µg/ml 4',6-Diamidino-2-phenylindole (DAPI), for fluorescence microscopy imaging. DIC and fluorescence images were acquired using a Zeiss Axio Observer.Z1 inverted microscope equipped with an AxioCam HRm Zeiss camera, with optical sectioning using using Zeiss ApoTome.2, Plan-Apochromat 63x/1.40 oil immersion objective, and the Zeiss ZEN 2 (blue edition) software. Z-stack images across the diameter of worms were captured in 500 nm intervals. The antibodies used were 1:1,000 rabbit anti-LMN-1, clone 3932 (generously provided by Prof. Yosef Gruenbaum); 1:500 mouse anti-β-Amyloid, 1-16 monoclonal antibody (6E10) (BioLegend Cat# 803001, RRID: AB_2564653); 1:200 Rhodamine Red-X-AffiniPure Donkey Anti-Rabbit IgG (Jackson ImmunoResearch Labs Cat# 711-295-152, RRID:AB_2340613); 1:200 Cy2-AffiniPure Donkey Anti-Mouse IgG (H+L) (Jackson ImmunoResearch Labs Cat# 715-225-151, RRID:AB_2340827).

RNA isolation

Total RNA was isolated using NucleoSpin® RNA kit (MACHEREY-NAGEL, 740955). For each time-point, 6,000 synchronized eggs were placed on NG-ampicillin plates seeded with HT115 bacteria and supplemented with IPTG. The worms were washed daily with M9 to discard of progeny. On each time-point, we washed the worms

with M9 to remove bacteria, left a minimal buffer volume, and froze them immediately in liquid nitrogen. The samples were thawed on ice, resuspended in 350 µl buffer RA1 (kit reagent) supplemental with 5.5 µl 1M DTT, and added a small amount of 0.5 mm zirconium oxide beads (Next Advance, ZrOB05). The worms were homogenized at 4 °C using a Bullet Blender® (Next Advance) set to speed eight, for three minutes, and then set to speed nine and for an additional two minutes. Homogenates were transferred to microcentrifuge tubes and centrifuged at 14,000 x g for 5 minutes. The supernatant was transferred to NucleoSpin® Filter (NucleoSpin® RNA kit reagent), and the next steps followed the manufacturer's standard protocol. Following RNA purification, the samples were stored at -80 °C.

Quantitative real-time PCR (qPCR)

Following RNA isolation, the RNA integrity was assessed by loading and running 1 µl of each sample in a 1% w/v SeaKem® LE Agarose (Lonza, 50004) gel supplemented with 1 µl ethidium bromide (Sigma-Aldrich, E1510) for a 40 ml gel, and visualizing the rRNA bands. The gels were run in an Owl™ EasyCast™ B1 Mini Gel Electrophoresis System (Thermo Scientific). The RNA purity and quantity were assessed by loading 1.5 µl of each sample onto a NanoDrop™ 2000c Spectrophotometer and reading the A_{260} nm emission, as well as the A_{260}/A_{280} and A_{260}/A_{230} ratios. cDNA was synthesized by reverse transcription from isolated RNA using the iScript cDNA synthesis kit (Bio-Rad, 170–8891). Each 20 µl reaction followed the manufacturer's instructions, with 1 µg of RNA template used per reaction. The reaction was incubated for 5 minutes at 25 °C, 20 minutes at 46 °C, and 1 min at 95 °C. The cDNA was stored at -20 °C. qPCR target information and the primer oligonucleotides sequences are detailed in Table S2.

qPCR was performed with iTaq™ Universal SYBR® Green Supermix (Bio-Rad, 172–5124). For usage in a qPCR reaction, each cDNA sample was diluted ten-fold, and to each 14 µl reaction, 1.4 µl of the diluted template was added. This corresponds to 700 pg of total RNA. 0.5 µM of forward and reverse primers were present in a reaction. The reactions were performed in technical replicates and read in a CFX96™ Real-Time PCR Detection System (Bio-Rad). Each reaction started with a polymerase activation and DNA denaturation for 30 sec 95 °C incubation, followed by 40 cycles of denaturation and annealing/extension, and plate read: 5 sec 95 °C, and 30 sec 60 °C, and finally 10 sec at 95 °C. For target specific, melting curve analysis was performed, in which the plate was read at 65°C up to 95 °C, at 5 sec 0.5 °C steps. Each reaction had a no-template control (NTC) included. The qPCR measurements were analyzed in Bio-Rad CFX Manager version 2.0.885.0923. Cq for all reactions was determined by a single threshold. The Cq levels were normalized to the geometric mean of three reference genes: *cdc-42*, *pmp-3,* and Y45F10D.4, which were selected based on past work (Hoogewijs, Houthoofd, Matthijssens, Vandesompele, & Vanfleteren, 2008), to generate ΔCq values. Relative expression levels were determined by the -ΔΔCq method, by calculating the difference from the mean ΔCq value of the untreated samples. Bar graphs show the relative expression values.

RNA sequencing (RNA-Seq)

Following RNA isolation, the RNA integrity and quantity was assessed using the Agilent 2200 TapeStation System. The RNA purity was assessed by loading 1.5 µl of each sample onto a NanoDrop™ 2000c Spectrophotometer and reading the A_{260} nm emission, as well as the A_{260}/A_{280} and A_{260}/A_{230} ratios. Isolated RNA samples were

prepared and sequenced using the Illumina HiSeq 2500 by the Technion Genome Center (Technion, Haifa, Israel) according to the CEL-Seq2 protocol, as described by its developers (Hashimshony et al., 2016). During CEL-Seq sample preparation, each sample is marked with a primer containing a unique barcode. Each initial RNA sample was barcoded using a pool of three different CEL-Seq primers. Using several primers to create technical replicates for each sample reduces the possibility of technical variability caused by differences in barcode efficiency. Processing was done with a modified CEL-Seq2 pipeline (yanailab.github.io/celseq2), based on the one described by its developers. Barcode splitting was done with their pipeline, with parameters: stats_file=stats.tab, min_bc_quality=10, bc_length=6, umi_length=6, cut_length=51. Adapter and quality trimming were performed using cutadapt ((Martin, 2011), cutadapt.readthedocs.io), version 1.12, with parameters: -e 0, -q 10, -u 9, -a "A{100}", -a "A{15}N{100}", -a GATCGTCGGACT, -n 3, -m 28. The set parameters mean no errors, trimming quality below 10 in a phred+33 scale, removing the first 9 bases, trimming adapters, poly A and poly N tails, and keeping only reads of length at least 28. Two sequences were set for removal: (1) "Small RNA 5' adapter" – GATCGTCGGACT; (2) Poly A, as during sample preparation a poly T sequence is used to capture the 3' end of the transcripts. After adapter and quality trimming over 90% of the reads remained. Mapping to the *C. elegans* genome, Ensembl: WBcel235, release 36 (June, 2017), joint with the ERCC sequences (CEL-Seq2 control Spike-In), was done using Tophat2 version 2.1.1, ((Kim et al., 2013), ccb.jhu.edu/software/tophat) with parameters: -N 2, --read-edit-dist 2, -G protein_coding_ERCC.gtf. The set parameters mean up to 2 mismatches, and up to an edit distance of 2, using annotations of protein coding genes (from Ensembl WBcel235

release 36), and the ERCC Spike-In. A mapping percentage of over 94% was reached. Gene counting was done using HTseq-count ((Anders, Pyl, & Huber, 2015), htseq.readthedocs.io) version 0.6.1, with parameters: gff_file=protein_coding_ERCC.gtf, umi=false. The set parameters mean that the counting was done using the same annotations as in the mapping step (protein coding and ERCC Spike-In) and ignoring UMIs. This was done since UMIs are too short to capture the full repertoire of transcripts in the samples and using them would cause a flattening of the signal for genes with a higher expression. Normalization and differential expression analysis done using the R/Bioconductor (Huber et al., 2015; R Core Team, 2018) package DESeq2 version 1.22.2 with default parameters (Love, Huber, & Anders, 2014). Genes with low expression (mean counts < 2) were discarded from the analysis. The similarity between replicates, and the relations between the samples, were evaluated by hierarchically clustering the Euclidean or the Pearson correlation distance measures of the samples and visualizing in heatmaps. Principal Component Analysis (PCA) plots were also used to ascertain the similarity between samples. The RNA sequencing data files that were generated in this study are available in the NCBI Gene Expression Omnibus (GEO) under the accession number GEO: GSE126585.

RNA-Seq results presentation and pathway analysis

Euler diagrams were generated using the R package eulerr version 5.1.0. GO Biological Processes *Gene Set Enrichment Analysis (GSEA)* was performed using the R/Bioconductor package fgsea version 1.8.0 (Sergushichev, 2016). KEGG GSEA was performed using the R/Bioconductor packages clusterProfiler version 3.8.1 (Yu, Wang, Han, & He, 2012) and org.Ce.eg.db version 3.6.0. RNA-Seq measured genes were ranked

by their log2 fold-change between control and treatment and were used to perform a GSEA in medium sized gene-sets (GO: 15-500 members, KEGG: greater than 15 members), using 10,000 permutations. *Protein Class overrepresentation* test was done using the online PANTHER classification system interface, set to perform a Binomial test followed by a Bonferroni correction (Released 2017-12-05), using the PANTHER database version 13.1 (2018-02-03), and the PANTHER Protein Class annotation data set (Thomas et al., 2003). The gene list that was selected to be tested for overrepresented protein classes, included all the genes whose expression fold-change passed the statistical significance criteria (p-value < 0.05). The reference/background list was composed of all the genes (20,057) in the PANTHER database for *C. elegans*.

Binding motif enrichment analysis

The presence of T-box transcription factor binding motifs in the promoter regions of genome-wide protein coding genes was determined using the R/Bioconductor package Biostrings version 2.50.0. The enrichment of T-box transcription factor binding motifs was determined using the R/Bioconductor package PWMEnrich version 4.16.0. The promoter regions of *C. elegans* genes were defined as the sequences between 500 bp upstream-, and 100 bp downstream, to the TSS, based on previous findings (Narasimhan et al., 2015; Niu et al., 2011). The promoter sequences were obtained using the R/Bioconductor package biomaRt version 2.38.0 (Durinck, Spellman, Birney, & Huber, 2009). The T-box transcription factors binding motifs were obtained from the CIS-BP database version 1.02 (Weirauch et al., 2014). The *Binding motifs heatmap* was composed using the R package pheatmap version 1.0.10. The genes that are presented in the heatmap possess at least one T-box transcription factor binding motifs, whose

presence was scored with a *p*-value < 0.01. The “Function” of selected genes is based on our interpretation of their Gene Ontology annotations. The genes were hierarchically clustered using the ‘hclust’ function with a binary distance measure set in the function ‘dist’.

Proteasome activity assay

For each treatment, 5,000 synchronized eggs were placed on NG-ampicillin plates seeded with HT115 bacteria and supplemented with IPTG. The worms were washed daily with M9 to discard of progeny. On day six of adulthood, the worms were washed with M9 to remove bacteria, and resuspended them twice in ice-cold PBS. Afterwards, a small amount of 0.5 mm zirconium oxide beads (Next Advance, ZrOB05) was added to the collected worms. The worms were homogenized at 4 °C using a Bullet Blender® (Next Advance) set to speed nine, three times for ten seconds each. Worm debris was removed by centrifuging at 780 x g for three minutes at 4 °C and transferring away the soluble upper post-debris fraction. The protein content of the post-debris fraction was quantified using the BCA Protein Assay Kit (Pierce™, 23227) according to its suggested protocol. Triplicate wells of an opaque 96-well plate (Thermo Scientific, Nunc F96 MicroWell, 137101) were each loaded with 10 µg of protein diluted in a 26S proteasome activity assay buffer (50 mM Tris-HCl pH 7.5, 40 mM potassium chloride, 5 mM magnesium chloride, 1 mM DTT, 0.5 mM ATP, 0.1% BSA). Each well had a total volume of 90 µl. 10 µl of 250 µM substrate was added to each well (2 M stock solution in DMSO), for a final concentration of 25 µM. The synthetic substrates used: chymotrypsin-like proteasome activity, Suc-LLVY-AMC (Enzo Life Sciences, BML-P802-0005); caspase-like activity, Z-LLE-AMC (Calbiochem, 539141); and trypsin-like activity, Z-ARR-

AMC (Calbiochem, 539149). The wells were excited at 380-nm and fluorescence emission was measured at 480-nm every five minutes for an hour at 37 ºC using an Infinite M200 PRO plate reader and Magellan version 7.1 (Tecan Trading AG, Switzerland). For each sample, identical triplicates were loaded with 100 µM MG132 (Sigma-Aldrich, M7449) and incubated for 5 minutes at room temperature prior to measurement as a control. Results at time 0 of each experiment were defined as baseline.

Statistical Analysis

Statistical details of experiments that include the statistical tests used, significance, error bars, repeats, exact values of n, and what they represent, can be found in the corresponding figure legends. Data are presented as mean ± SEM. Statistical analyses were performed using GraphPad Prism version 7.01 for Windows, GraphPad Software, La Jolla California USA, www.graphpad.com, and R version 3.5.1 (R Core Team, 2018). p-values are indicated by ∗ for $p < 0.05$, ∗∗ for $p < 0.01$, ∗∗∗ for $p < 0.001$, **** for $p < 0.0001$, and “ns" for $p > 0.05$. “Statistically significant” was defined as p-value < 0.05.

References

Anders, S., Pyl, P. T., & Huber, W. (2015). HTSeq--a Python framework to work with high-throughput sequencing data. *Bioinformatics (Oxford, England)*, *31*(2), 166–169. https://doi.org/10.1093/bioinformatics/btu638

Durinck, S., Spellman, P. T., Birney, E., & Huber, W. (2009). Mapping identifiers for the integration of genomic datasets with the R/Bioconductor package biomaRt. *Nature Protocols*, *4*(8), 1184–1191. https://doi.org/10.1038/nprot.2009.97

Haithcock, E., Dayani, Y., Neufeld, E., Zahand, A. J., Feinstein, N., Mattout, A., … Liu, J. (2005). Age-related changes of nuclear architecture in Caenorhabditis elegans. *Proceedings of the National Academy of Sciences of the United States of America*, *102*(46), 16690–16695. https://doi.org/10.1073/pnas.0506955102

Hakim, A., Mor, Y., Toker, I. A., Levine, A., Neuhof, M., Markovitz, Y., & Rechavi, O. (2018). WorMachine: machine learning-based phenotypic analysis tool for worms. *BMC Biology*, *16*(1), 8. https://doi.org/10.1186/s12915-017-0477-0

Hashimshony, T., Senderovich, N., Avital, G., Klochendler, A., de Leeuw, Y., Anavy, L., … Yanai, I. (2016). CEL-Seq2: Sensitive highly-multiplexed single-cell RNA-Seq. *Genome Biology*, *17*(1), 1–7. https://doi.org/10.1186/s13059-016-0938-8

Holmberg, M., & Nollen, E. A. A. (2013). Analyzing modifiers of protein aggregation in C. elegans by native agarose gel electrophoresis. *Methods in Molecular Biology (Clifton, N.J.)*, *1017*, 193–199. https://doi.org/10.1007/978-1-62703-438-8_14

Hoogewijs, D., Houthoofd, K., Matthijssens, F., Vandesompele, J., & Vanfleteren, J. R. (2008). Selection and validation of a set of reliable reference genes for quantitative sod gene expression analysis in C. elegans. *BMC Molecular Biology*, *9*, 9.

https://doi.org/10.1186/1471-2199-9-9

Huber, W., Carey, V. J., Gentleman, R., Anders, S., Carlson, M., Carvalho, B. S., … Morgan, M. (2015). Orchestrating high-throughput genomic analysis with Bioconductor. *Nature Methods*, *12*(2), 115–121. https://doi.org/10.1038/nmeth.3252

Irianto, J., Lee, D. A., & Knight, M. M. (2014). Quantification of chromatin condensation level by image processing. *Medical Engineering and Physics*, *36*(3), 412–417. https://doi.org/10.1016/j.medengphy.2013.09.006

Kim, D., Pertea, G., Trapnell, C., Pimentel, H., Kelley, R., & Salzberg, S. L. (2013). TopHat2: accurate alignment of transcriptomes in the presence of insertions, deletions and gene fusions. *Genome Biology*, *14*(4), R36. https://doi.org/10.1186/gb-2013-14-4-r36

Love, M. I., Huber, W., & Anders, S. (2014). Moderated estimation of fold change and dispersion for RNA-seq data with DESeq2. *Genome Biology*, *15*(12), 550. https://doi.org/10.1186/s13059-014-0550-8

Martin, M. (2011). Cutadapt removes adapter sequences from high-throughput sequencing reads. *EMBnet.Journal*, *17*(1), 10–12. https://doi.org/http://dx.doi.org/10.14806/ej.17.1.200

Narasimhan, K., Lambert, S. A., Yang, A. W., Riddell, J., Mnaimneh, S., Zheng, H., … Hughes, T. R. (2015). Mapping and analysis of Caenorhabditis elegans transcription factor sequence specificities. *ELife*, *4*, e06967. https://doi.org/10.7554/eLife.06967

Niu, W., Lu, Z. J., Zhong, M., Sarov, M., Murray, J. I., Brdlik, C. M., … Reinke, V. (2011). Diverse transcription factor binding features revealed by genome-wide ChIP-seq in C. elegans. *Genome Research*, *21*(2), 245–254.

https://doi.org/10.1101/gr.114587.110

R Core Team. (2018). R: A Language and Environment for Statistical Computing. Vienna, Austria. Retrieved from https://www.r-project.org/

Sergushichev, A. (2016). An algorithm for fast preranked gene set enrichment analysis using cumulative statistic calculation. *BioRxiv*, 60012. https://doi.org/10.1101/060012

Sosnik, J., Vieira, W. A., Webster, K. A., Siegfried, K. R., & McCusker, C. D. (2017). A new and improved algorithm for the quantification of chromatin condensation from microscopic data shows decreased chromatin condensation in regenerating axolotl limb cells. *PLoS ONE*, *12*(10), 1–12. https://doi.org/10.1371/journal.pone.0185292

Stiernagle, T. (2006). Maintenance of C. elegans. *WormBook : The Online Review of C. Elegans Biology*, 1–11. https://doi.org/10.1895/wormbook.1.101.1

Thomas, P. D., Campbell, M. J., Kejariwal, A., Mi, H., Karlak, B., Daverman, R., … Narechania, A. (2003). PANTHER: a library of protein families and subfamilies indexed by function. *Genome Research*, *13*(9), 2129–2141. https://doi.org/10.1101/gr.772403

Weirauch, M. T., Yang, A., Albu, M., Cote, A. G., Montenegro-Montero, A., Drewe, P., … Hughes, T. R. (2014). Determination and inference of eukaryotic transcription factor sequence specificity. *Cell*, *158*(6), 1431–1443. https://doi.org/10.1016/j.cell.2014.08.009

Yu, G., Wang, L.-G., Han, Y., & He, Q.-Y. (2012). clusterProfiler: an R package for comparing biological themes among gene clusters. *Omics : A Journal of Integrative Biology*, *16*(5), 284–287. https://doi.org/10.1089/omi.2011.0118
